# Greenspace exposure and associated health outcomes: a systematic review of reviews

**DOI:** 10.12688/f1000research.148878.2

**Published:** 2025-07-30

**Authors:** Brittnee Bryer, Chinonso Christian Odebeatu, Wen Ray Lee, Kathryn Vitangcol, Victor Gallegos-Rejas, Nicholas J. Osborne, Gail Williams, Darsy Darssan

**Affiliations:** 1 School of Public Health, The University of Queensland, Herston, Queensland, Australia; 2 Child Health Research Centre, The University of Queensland, South Brisbane, Queensland, Australia; 3 Princess Alexandra Hospital Southside Clinical Unit, The University of Queensland, Woolloongabba, Queensland, Australia; 4 Centre for Online Health, The University of Queensland, Woolloongabba, Queensland, Australia; 5 Centre for Health Services Research, The University of Queensland, Woolloongabba, Queensland, Australia; 6 School of Population Health, University of New South Wales, Sydney, New South Wales, Australia; 7 European Centre for Environment and Human Health, University of Exeter, Truro, UK

**Keywords:** Human health, natural environment, outdoor environment, umbrella review, biodiversity, greenery, urbanisation

## Abstract

**Background:**

Numerous systematic reviews and meta-analyses have sought to clarify the relationship between greenspace exposure and health outcomes, but the results are inconsistent. We aimed to synthesise all relevant systematic reviews and meta-analyses on this association.

**Methods:**

We searched five databases (PubMed, Embase, the Cumulative Index to Nursing and Allied Health Literature [CINAHL], Scopus, and the Cochrane Database of Systematic Reviews) and conducted a manual reference search for systematic reviews and meta-analyses written in English and published in peer-reviewed journals that clearly defined measures of greenspace exposure and reported health outcomes directly attributable to greenspace exposure. A total of 36 systematic reviews published between January 2010 and December 2020 were identified for inclusion in this systematic review of reviews (PROSPERO: CRD42021227422). The methodological quality and risk of bias of included systematic reviews were evaluated by two independent reviewers.

**Results:**

Beneficial effects of greenspace exposure were observed for all-cause and cause-specific mortality, as well as mental health and cognitive function. Ambivalent results were found for cardiovascular and metabolic health, general health and quality of life (QOL), and respiratory health and allergies. Most of the systematic reviews included in the current umbrella review had a low to moderate methodological quality and a high risk of bias.

**Conclusions:**

This umbrella review highlights the link between greenspaces and a variety of health outcomes, emphasising the importance of preserving existing greenspaces and integrating additional vegetation into urban areas to maintain public health.

## Introduction

Reducing the global burden of diseases has become a public health priority, and many international bodies, including the World Health Organisation (WHO), have highlighted the need to maintain healthy, liveable, and sustainable cities ( World Health Organization, 2021). In response to this, changes to urban environmental design, such as the provision of access to greenspace exposure, have been proposed to enhance human health and wellbeing ( Browning et al., 2022; Nguyen et al., 2021).

Greenspace may benefit human health via several mechanisms, including increased physical activity (PA), enhanced social engagement, and improved mental restoration ( Zhang et al., 2022). Accumulating evidence from the past two decades has suggested that exposure to greenspace may reduce the risk of all-cause and cause-specific mortality and morbidity ( Rojas-Rueda et al., 2019; Twohig-Bennett & Jones, 2018; Yuan et al., 2021), and birth outcomes ( Dzhambov et al., 2014; Lee et al., 2020; Twohig-Bennett & Jones, 2018). However, several systematic reviews did not report statistically significant findings between greenspace and health outcomes, particularly for respiratory diseases in children and adolescents ( Lambert et al., 2017), and the risk of cancer in adults ( Porcherie et al., 2021). In contrast, a study carried out in Australia found that the odds of skin cancer escalate in accordance with increased neighbourhood greenness ( Astell-Burt et al., 2014). These inconsistencies could be attributed, in part, to heterogeneity across different outcome measures, inconsistent definitions of greenspace exposure, or disparate study designs and confounding variables. Despite this discordance, exposure to greenspace may have beneficial effects on health outcomes.

There is an increasing number of systematic reviews and meta-analyses on the relationship between greenspace exposure and health outcomes ( Houlden et al., 2018; Lambert et al., 2017; Lee et al., 2020; Rojas-Rueda et al., 2019; Twohig-Bennett & Jones, 2018; Yuan et al., 2021). However, many of these reviews only consider a narrow selection of health outcomes. Therefore, the literature needs to be assessed to understand the association between greenspace exposure and health outcomes, excluding health determinants and the influence of blue space. Addressing this gap requires a higher level of critical appraisal and synthesis of all systematic reviews and meta-analyses. Only two umbrella reviews have reviewed evidence on the link between exposure to greenspace and human health from systematic reviews and meta-analyses ( van den Bosch & Ode Sang, 2017; Yang et al., 2021). The review by van den Bosch and Ode Sang (2017) focused on the natural environment, including both green and blue spaces. The conclusions drawn do not isolate the impact of greenspace exposure alone on health outcomes, as its effects are confounded by blue space. Yang et al. (2021) evaluated the combined effects of health determinants (i.e., physical activity, time spent outdoors, prosocial behaviour, and academic achievement) and health outcomes, obscuring the isolated impact of greenspace exposure on health outcomes alone. Yang et al. (2021) also included scoping reviews in their synthesis.

There is currently no umbrella review in the literature that possesses all the following qualities: i) evaluates the association between greenspace exposure and health outcomes from quantitative studies, independently of blue space and health determinants; ii) assesses both the quality and risk of bias of reviews; iii) records the frequency of greenspace exposure measure used; iv) summarises health outcomes and associated ICD-10 codes; v) reports effect measures (e.g. odds ratio [OR], mean difference [MD]) from meta-analyses. This umbrella review aims to fill this gap.

## Methods

The protocol for this umbrella review was registered with the International Prospective Register of Systematic Reviews (PROSPERO) (registration ID: CRD42021227422) ( Gallegos-Rejas et al., 2021).

### Search strategy

An electronic literature search was carried out using the following databases: PubMed, Embase, the Cumulative Index to Nursing and Allied Health Literature (CINAHL), Scopus, and the Cochrane Database of Systematic Reviews. We restricted the search to peer-reviewed systematic reviews and meta-analyses conducted in humans and published in English between January 2010 and December 2020, including reviews first published online during this period (extended data: Supplementary Table S1). All databases were searched on 05/01/2021. To ensure that relevant systematic reviews were retrieved, we also conducted forward and backward reference searching on screened reviews, as well as manual searching of reference lists of relevant reviews. EndNote software programme was used for reference management ( The EndNote Team, 2013).

### Eligibility criteria

Title and abstract screening and full-text screening of identified systematic reviews were independently conducted by two authors. The following inclusion criteria were used: i) systematic review or meta-analysis; ii) written in English and published in a peer-reviewed journal; iii) greenspace exposure clearly defined using objective or subjective measures; iv) reported health outcome(s) were directly attributable to greenspace exposure. We excluded: i) systematic reviews that failed to follow a standardised, systematic review approach, for example, no mention of databases searched; no identification of the search terms used; or no assessment of original studies quality; ii) scoping reviews; iii) systematic reviews that did not consider the effects of greenspace independently of blue space. Reviews that mentioned health outcome(s) in the title and abstract but only reported health determinants such as body mass index (BMI), physical activity (PA), or diet were excluded during full-text screening (extended data: Supplementary File S1). Disagreements were resolved via discussions with the last author. Covidence was used to conduct all stages of study screening ( Covidence systematic review software, 2022).

### Data extraction

Data extraction was conducted independently by two authors. Pilot testing was conducted on 10 of the identified systematic reviews to build the data extraction form. The form included information on the design of original studies, types and measures of greenspace exposures, and types of health outcomes. Any disagreement in the data extraction process was resolved via discussions with the last author.

### Analysis criteria

The objectives, methods, and conclusions of each systematic review included in this umbrella review were summarised. Quantitative data, reported as the effect estimate with associated confidence interval, were summarised for health outcomes if available. We could not conduct a meta-analysis as the primary aim of any umbrella review is to summarise existing systematic reviews and not to re-synthesise or conduct meta-analysis ( Aromataris et al., 2015).

The number and types of original studies in the systematic reviews, as well as the measure(s) of greenspace exposure and health outcomes, were recorded. The International Classification of Diseases, 10 ^th^ Edition (ICD-10) was then used to allocate corresponding ICD-10 codes for each health outcome identified during data extraction ( World Health Organization, 2019).

The methodological quality and risk of bias of included systematic reviews were evaluated independently by two authors using A MeaSurement Tool to Assess systematic Reviews 2 (AMSTAR-2) ( Shea et al., 2017) and the Risk of Bias Assessment Tool for Systematic Reviews (ROBIS) ( Whiting et al., 2016), respectively. Disagreements between researchers were solved via discussions with the last author. No systematic reviews were excluded based on the methodological quality or risk of bias assessment.

## Results

### Study selection

Our initial systematic search yielded 4250 systematic reviews. After removing duplicates and screening titles and abstracts, 173 potentially relevant articles were selected for full-text assessment. After full-text evaluation, we excluded a total of 141 articles based on our inclusion criteria and retained a total of 36 systematic reviews. The PRISMA flowchart is available under the Reporting guidelines section at the end of this article.

### Study type and characteristics

Of the 36 systematic reviews included in this umbrella review, most (n=29, 75%) were published after 2016, with only seven (25%) published before or during 2016. The number of original studies included in systematic reviews ranged from 5 to 143 ( Table 1), totalling 989. The majority of these were cross-sectional (n=418, 42.7%), followed by cohort (n=101, 10.3%), longitudinal studies (n=64, 6.5%), RCTs (n=61, 6.2%), and case-control studies (n=5, 0.5%) ( Table 2).

**Table 1.** Characteristics of included systematic reviews.

Author (year)	Total number of original studies	Type of study (n)	Objective(s) of the study	Sample size	Greenspace measures	Outcome measures	Main finding(s)
Akaraci et al. (2020)	37	Cross-sectional (30), cohort (4), case-control (2), quasi-experimental (1)	To systematically review and synthesise (meta-analyse) studies on the impacts of green and/or blue spaces on birth outcomes.	427-3,026,603	NDVI within several buffer distances, increment of 10% green space measures, residential greenness following a 0.1 unit increase in NDVI.	BW, PTB, SGA, LBW.	A statistically significant relationship was found between increased residential greenness and higher BW and lower odds of SGA. Non-significant associations reported for LBW and PTB outcomes.
Carver et al. (2020)	9	Observational (5), Quasi-experimental (4)	To examine existing evidence on the association between availability and use of greenery and mental health among residents of aged care facilities.	10-211	Garden exposure/garden use.	Wellbeing, stress, depression.	Exposure to greenery and use of greenspace in residential aged care facilities show promise for promoting mental health. However, the findings relied mainly on non-validated measures of mental health.
de Keijzer et al. (2020)	59	Cross-sectional (44), longitudinal (14), ecological (1)	To systematically review existing evidence on the relationship of long-term outdoor greenspace exposure with healthy ageing.	80-429,334	NDVI within a certain buffer distance, percentage/proportion of greenspace/parklands/tree canopy/vegetation within a buffer, proximity/distance to greenspace.	Mental health, cognitive function, physical capability, morbidity, weight status, hypertension, cholesterol, perceived wellbeing.	Suggestive salutogenic effects of increased long-term exposure to greenspace on healthy ageing, but evidence base is limited/inadequate to draw conclusion.
de Keijzer et al. (2016)	13	Cross-sectional (8), longitudinal (3), ecological (2)	To systematically examine the link between long-term greenspace exposure and cognition over life span.	17-2805	Neighbourhood measures of percentage of green space, greenness surrounding schools, NDVI, rating of greenness, self-reported or views of greenspace through the window.	Cognitive function, dementia.	The association between greenspace exposure and cognition were inadequate but suggestive of a positive relationship.
Dzhambov et al. (2014)	8	Cross-sectional (8)	To explore the association between pregnant women living in green environments and BW of their infants.	2393-81,246	Residential greenness within a 100m buffer.	BW	A weak but positive association between residential greenness within a 100 m buffer and BW.
Gascon et al. (2016)	12	Ecological (7), Cohort (3), cross-sectional (2)	To systematically synthesise the existing evidence on the relationship between residential natural outdoor environments and mortality in adults.	1645-43,000,000	10% increase in residential greenness (determined via percentage greenspace in an area or NDVI), interquartile range increase as a proxy for higher vs lower greenspace exposure categories.	All-cause mortality, CVD mortality, lung cancer, respiratory diseases, diabetes mortality, intentional self-harm, motor vehicle fatality.	Inconsistent evidence between higher residential greenness and all-cause mortality, however the risk of CVD mortality was reduced following increased exposure to residential greenness.
Gascon et al. (2015)	28	Cross-sectional (21), longitudinal (6), ecological (1)	To systematically review the long-term effects of residential green spaces on mental health.	~100-345,143	Surrounding greenness, access to green spaces, quality of green spaces.	General mental health, stress, distress, depression, anxiety, mood disorders, emotional and behavioural health.	Limited evidence of positive association of surrounding greenness, access to green space, and quality of greenspace with mental health benefits.
Gritzka et al. (2020)	10	RCT (8), preliminary cross-sectional survey (1), acute RCT (1)	To systematically evaluate existing research on mental health and wellbeing outcomes on employees participating in nature-based interventions.	14-94	Green exercise, nature savouring, green office space.	Mental health, cognitive ability, recovery and restoration, work and life satisfaction, psychophysiological indicators of health.	Nature-based interventions had a positive impact on employees’ mental health indices. The effect was less consistent for other health outcomes.
Hartley et al. (2020)	7	Cross-sectional (4), cohort (2), meta-analysis (1)	To examine existing evidence on the relationship between greenness and asthma in children.	150-49,956	NDVI, land-use classification, total and native land-use types,	Childhood asthma.	Higher greenness had little or no impact on childhood asthma.
Houlden et al. (2018)	52	Cross-sectional (37), longitudinal (6), case control (4), uncontrolled case (4), ecological (1)	To systematically synthesise evidence on greenspace and mental wellbeing.	32-65,407	Amount of local-area greenspace, greenspace types, views of greenspace, greenspace accessibility, subjective connection to nature.	Hedonic wellbeing (happiness and life satisfaction), eudaimonic wellbeing (fulfilment, functioning and QoL). Psychological distress, vitality, mood, attention affect.	Relationship between the amount of local-area greenspace and hedonic but not eudaimonic wellbeing supported. Limited evidence observed for other greenspace measure.
Islam et al. (2020)	23	Cross-sectional (11), prospective cohort (10), unknown (2)	To examine existing evidence on the association between greenspace exposure and early childhood development.	253-3,026,603	Surrounding/residential greenness, distance to city parks.	BW, PTB, gestational age, atopic dermatitis, PA, BMI, neuro-behavioural health, asthma, bronchitis, wheezing.	Increased greenspace exposure during pregnancy was associated with decreased LBW. The risk of obesity and neurodevelopmental issues such as inattentiveness were reduced following increased exposure to greenness. Certain green plants may exacerbate asthma symptoms in children.
Kabisch et al. (2017)	27	NA	To examine the state of evidence on the association between urban green spaces and the health of children and older adults in urbanised areas.	NA	Average NDVI, urban land-use data, tree coverage based on satellite data, public parks and playground space, percentage of greenery from census block or land-use data, urban allotment garden.	Children’s health: infant/neonatal mortality, BW, mental health, weight status, allergic sensitisation, asthma. Elderly health: CVD mortality, respiratory mortality, diabetes, mental health, general wellbeing, cancer, respiratory diseases.	Urban greenspaces offer protective effects on children and elderly health, but the evidence base is inconclusive and may be driven by socioeconomic confounders.
Kondo et al. (2018)	68	Experimental (35), longitudinal (20), quasi-experimental (9), case-crossover (3), RCT (1)	To systematically synthesise existing evidence examining the association between urban green space and human health.	12-3,026,603	Average NDVI within proximity of residence, percentage of greenspace within administrative boundary of residence, distance from residence to nearest park or greenspace, percentage of natural spaces and parks near home, quantity of neighbourhood green space.	BW, PTB, SGA, prostate cancer, weight status, BMI, cortisol, diabetes, cardiovascular health, mental health, PA, violence/aggression, behavioural problems.	While urban greenspace exposure was associated with mortality, violence, attention, and mood, mixed or no association results were reported for weight status, depression, stress, and general health. Limited evidence to draw conclusions on BW, cancer, diabetes, and respiratory symptoms.
Lachowycz and Jones (2011)	60	NA	To systematically examine the link between greenspace access and obesity, obesity-related outcomes, and behaviour.	58-345,143	Distance to or count of nearest greenspace from home, percentage greenspace, audit of greenness, quality of greenspace, type of greenspace, park service areas.	Obesity, CHD, diabetes, premature mortality, circulatory disease, MetS, weight status, PA.	Weak but positive association between greenspace and obesity-related health indicators. The relationship varied based on age, socio-economic status, and greenspace measure.
Lambert et al. (2018)	5	Cohort (5)	To synthesise current literature on the relationship of surrounding greenness with atopic sensitisation in children and adolescents.	94-13,016	NDVI, land-cover database, LiDAR imagery.	Atopic sensitisation.	Findings were mixed with some cohorts showing protective effects from greenspace, while others reported an adverse or no effect following greenspace exposure.
Lambert et al. (2017)	11	Cohort (5), ecological (3), case-control (2), cross-sectional (1)	To systematically evaluate the relationship between surrounding residential greenness and allergic respiratory diseases in children and adolescents.	549-642,313	NDVI, street tree density, LiDAR.	Asthma, allergic rhinitis.	Relationship between residential greenness and asthmas and allergic rhinitis remains inconclusive. Pooled estimate from meta-analysis showed no significant association.
Lee et al. (2020)	10	NA	To investigate the link between pregnancy outcomes and the surrounding living environment such as greenness.	2393-780,435	NDVI within 100, 250, and 500m buffers from participant’s home location.	LBW, very LBW, SGA, PTD, very PTD.	Weak evidence of positive association found between surrounding greenness and BW, with significant decrease in the incidence of LBW, SBA, and PTD.
Luo et al. (2020)	57	Cross-sectional (46), cohort (11)	To synthesise epidemiological studies on the relationship of greenspace exposure with overweight/obesity.	102-97,574,613	NDVI, residential proximity to the nearest greenspace, proportion of greenspace within 30 to 1600m buffer around residential address, number of parks, other greenery and shrub density.	Weight status.	Over half of the included studies reported beneficial relationship of greenspace with overweight/obesity. Increased NDVI was associated with overweight/obesity but not with proximity to greenspace, proportion of greenspace or number of parks in the meta-analysis.
Oh et al. (2017)	6	RCT (6)	To evaluate existing evidence on the health effects of spending time in natural green environments.	18-99	Mountain forest walking, 9-day forest healing camp.	Hypertension, cortisol, immune function, anxiety and depression, mood, BP, HR, inflammation, cardiac and pulmonary function, oxidative stress.	Natural environment had positive effects on physiological (hypertension, stress hormone, and immune function), and psychological (anxiety and depression) responses. However, all included studies had a high risk of bias.
Roberts et al. (2019)	33	Randomised crossover (16), non-randomised crossover (5), other (12)	To summarise and critically synthesise previous evidence on the impacts of short-term exposure to the natural environment on depressive mood.	8-280	Exposure to high levels of unmodified greenery.	Depression, affect, mood.	Short-term exposure to the natural environments led to a reduction in depressive mood, though the effects is only minimal, and included studies were of low quality and highly biased.
Rojas-Rueda et al. (2019)	9	Cohort (9)	To meta-analyse longitudinal studies on the exposure-response function between green spaces and all-cause mortality.	1645-4,284,680	Interquartile range or 0.1 unit increase in NDVI within 500m or less from the participant’s home location.	All-cause mortality.	A significant inverse association found between increased residential greenness and all-cause mortality.
Schulz et al. (2018)	18	Cross-sectional (14), longitudinal (4)	To systematically review empirical studies on the association between the built environment and mortality/morbidity in Germany.	2001-20,000	Exposure to natural or human-built areas which are covered with grass, trees, shrubs, and other vegetations.	Overall health, mental health, T2DM, cancer mortality, CVD mortality, acute respiratory illness, chronic/allergic illnesses.	No association was found for chronic respiratory conditions, however acute respiratory symptoms appeared to be associated with higher greenery. Other health outcomes such as diabetes, cancer, and CVD were rarely studied in the German built environment.
Shaffee and Abd Shukor (2018)	42	NA	To examine the association between greenspaces and stress reduction.	NA	Exposure to tended greenspace, forests, specific landscape elements, restorative landscape characteristics, and nature sounds.	Stress.	Nature settings were significantly but independently associated with human stress reduction in most included studies.
Shuda et al. (2020)	12	Cross-sectional (8), RCT (4).	To systematically explore the impacts of nature exposure on physiological markers and perceived stress.	32-4338	Average NDVI, percent tree canopy, percentage of greenspace, access to garden, active use of greenspace.	Perceived stress, salivary cortisol, BP, HRV.	Increased exposure to nature led to a decrease in various physiological markers of stress in most included studies. Similar effects were shown for perceived stress.
Shuvo et al. (2020)	22	Cross-sectional (14), observational (8)	To examine studies looking into the health effects of urban greenspace in low- and middle-income countries.	NA	Perceived frequency and duration of using urban greenspace, quality of urban greenspace, self-reported distance to nearest urban greenspace, proximity to and density of urban greenspace.	General mental health, anxiety, weight status, physical wellbeing, general health, QoL.	Compared to subjective measures, objective measures of greenspace had a modest relationship with the identified health outcomes.
Soga et al. (2017)	21	Case study (21)	To meta-analyse available evidence on the impacts of gardening on health.	14-514	Gardening types (horticultural therapy, daily gardening, and experimental short-term gardening).	Depression, cognitive function, positive affect, anxiety, stress, mood, QoL, social health, general health, self-esteem, psychological wellbeing and hope, BMD, loneliness, life satisfaction, anger, fatigue, confusion, tension, BMI, vigour.	Engaging in gardening is potentially beneficial to a range of health outcomes including reduction of depression, stress, and anxiety.
Twohig-Bennett and Jones (2018)	143	Cross-sectional (69), cohort (35), ecological (18), intervention (40)	To systematically review studies on the association between greenspace exposure and a range of health outcomes.	9 - >63,000,000	Neighbourhood greenspace, greenspace-based intervention, proximity to large greenspaces, NDVI, land cover map, tree canopy, street tree data, self-reported neighbourhood greenspace quality, self-reported walking in a green area, viewing trees through hospital window.	Cortisol, HR, cholesterol, BP, DBP, gestational age, PTB, SGA, stroke, T2DM, hypertension, CVD mortality, asthma, CHD, all-cause mortality.	Findings from meta-analysis showed a decreased risk in PTB birth, T2DM, CVD mortality, stroke incidence, hypertension, CHD, and all-cause mortality following increased exposure to greenspace.
Vanaken and Danckaerts (2018)	21	Cross-sectional (12), longitudinal (7), ecological (2).	To determine whether there is a relationship between exposure to greenspace and children’s and adolescents’ mental health and neurocognitive development.	72 - ~3,000,000	Land cover map, NDVI data, geolocation data, self-reported time spent in or distance to greenspaces.	Emotional and behavioural problems, mental wellbeing, neurocognitive development.	Consistent salutogenic relationship between exposure to greenspace and children’s emotional and behavioural issues, notably with inattention and hyperactivity challenges. Evidence related to children’s mental health, including depressive symptoms, were less clear.
van den Berg et al. (2015)	40	Cross-sectional (35), longitudinal (5)	While assessing the methodological quality, the study aimed to examine the link between perceived general and mental health, and all-cause mortality and the amount and quality of greenspaces.	NA	Percentage of greenspace, NDVI, distance to nearest greenspace or parks, presence of green qualities, presence of a private garden, observation of visible green elements in streets.	Perceived general health, perceived mental health, all-cause mortality.	Significant positive relationships between objectively measured greenspace around participant’s home and perceived mental health and all-cause mortality. Modest evidence was found for perceived general health.
Wen et al. (2019)	28	RCT (17), non-RCT (11)	To explore the relationship between the forest environment and human health while assessing the methodological quality of individual studies.	4-128	Exposure to forest environment.	Physical health, psychological health.	Improvement in physical and psychological health was observed following forest bathing
Whear et al. (2014)	17	Pre-post (6), prospective cohort (1), RCT (2), qualitative (7), mixed methods (1)	To explore the effects of gardens and outdoor spaces on mental and physical well-being of individuals with dementia residing in care homes.	NA	Use, view or experience of gardens, horticulture therapy.	Dementia-related behaviours, affect, time spent sleeping, quality of sleep, PA, medication use.	Garden exposure had some beneficial impacts. Evidence suggested garden use was associated with reduced agitation.
Yeo et al. (2019)	26	RCT (3), cluster RCT (4), controlled clinical trial (8), crossover study (3), one-group design (8)	To synthesise the health effects of indoor nature exposure for elderly individuals in residential settings.	10-85	Active nature programs via indoor gardening, horticulture activities, or horticulture therapy. Passive nature intervention such as indoor gardens, nature corridor enhancement, aquariums, and nature photos.	Dementia-related outcomes, psychological wellbeing, social health, functional and physical health, physiological health, general health, wellbeing and satisfaction.	Modest evidence that indoor nature exposure may improve the health and wellbeing of older adults in residential care, but most included studies were of low quality and prone to bias.
Yuan et al. (2021) *	22	Cohort (17), cross-sectional (5)	To synthesise evidence on the association between mortality and cardiovascular outcomes in older adults following greenspace exposure.	1084-5,988,606	NDVI, comparison of highest and lowest greenness exposure category.	All-cause mortality, CVD.	Most studies found a reduced risk of all-cause mortality and total CVD with increased greenness. Meta-analysis showed similar effects, even with stroke mortality.
Zhan et al. (2020)	36	Cross-sectional (19), cohorts (14), case-control (2), ecological (1)	To examine dose-response relationship of residential greenness with adverse pregnancy outcomes.	427-6,567,580	NDVI, proximity to greenspace, distance to nearest greenspace, percentage of greenspace.	BW, LBW, SGA, PTB, gestational age, head circumference.	Participants exposed to the highest level of greenery experienced higher BW. Higher greenness led to a reduced odds of LBW and SGA. A 0.1 unit increase in NDVI within 300m buffer led to a 2% decreased risk of LBW. No association was found for PTB or gestational age.
Zhang et al. (2020)	14	Cross-sectional (10), controlled experiment (3), longitudinal (1)	To investigate the link between greenspace exposure and adolescents’ mental wellbeing.	60-17,249	NDVI, percentage of neighbourhood greenspace, percentage of land use, perceived greenery, percentage of total land cover.	Stress, mood, depression, emotional wellbeing, mental health behaviours, psychological distress.	Suggests salutogenic effects of greenspace exposure on reduction of stress, depressive symptoms, and psychological distress, as well as better emotional wellbeing, and positive mood.
Zhang et al. (2017)	27	Cross-sectional (5), case series (3), RCT (3), quantitative descriptive (2), non-RCT (1), qualitative (3), phenomenology (5), case study (2), triangular (2) embedded (1)	To systematically evaluate the health benefits of the use and design of natural environments for people with mobility impairments.	1-1010	Activities carried out within the context of the natural environment, including passive involvement, active interventions, and rehabilitative interventions.	Physical health, mental health, social health.	Exposure to natural environment provided mental health benefits.

BMD: bone mineral density; BMI: body mass index; BP: blood pressure; BW: birthweight; CHD: coronary heart disease; CVD: cardiovascular disease; DBP: diastolic blood pressure; HR: heart rate; HRV: heart rate variability; LBW: low birth weight; LiDAR: light detection and ranging; NA: not available; MetS: metabolic syndrome; NDVI: normalised difference vegetation index; PA: physical activity; PTB: preterm birth; QoL: quality of life; RCT: randomised controlled trial; SGA: small for gestational age; T2DM: type 2 diabetes mellitus.

* First published online September 2020.

**Table 2.** Study design of original studies in included systematic reviews.

Study Design	n (%)
Cross-sectional	418 (42.7%)
Cohort	101 (10.3%)
Longitudinal	64 (6.5%)
Randomised Controlled Trial (RCT)	61 (6.2%)
Case-control	5 (0.5%)
Other ^a^	331 (33.8%)

^a^ ‘Other’: acute RCT, case study, case-crossover, case-series, controlled clinical trial, controlled case study, crossover, crossover trial, cross-sectional and observational, ecological, embedded design, experimental, factorial, field, interventional, interview, lab experiment, meta-analysis, mix methods, natural experiment, non-RCT, observational, one group, parallel groups, phenomenology, pre-post study, qualitative, quantitative, quasi-experiment, survey, systematic review, triangulation design, uncontrolled case study.

### Greenspace exposure

Measures of greenspace exposure varied significantly between systematic reviews. Of the 36 systematic reviews included in this umbrella review, 22 (61%) reported more than one greenspace measure ( Table 3). The normalised difference vegetative index (NDVI) was the most common measure of greenspace, used in 21 systematic reviews (58%). This was followed by proximity to greenspace (n=12, 33%), land cover map (n=8, 22%), duration of stay in greenspace setting (n=7, 19%), quality of greenspace (n=6, 17%), tree canopy (n=5, 14%) and frequency of greenspace usage (n=4, 11%) ( Table 3).

**Table 3.** Measures of greenspace exposure in included systematic reviews.

Greenspace Exposure Measure	Counts of Greenspace Exposure Measure (% out of 36 reviews)	Review Articles
NDVI ^a^	21 (58.8%)	Akaraci et al. (2020); de Keijzer et al. (2020); de Keijzer et al. (2016); Dzhambov et al. (2014); Gascon et al. (2015); Gascon et al. (2016); Hartley et al. (2020); Islam et al. (2020); Kabisch et al. (2017); Kondo et al. (2018); Lambert et al. (2017); Lambert et al. (2018); Lee et al. (2020); Luo et al. (2020); Rojas-Rueda et al. (2019); Shuda et al. (2020); Twohig-Bennett and Jones (2018); van den Berg et al. (2015); Vanaken and Danckaerts (2018); Yuan et al. (2021); Zhan et al. (2020)
Proximity to greenspace	12 (33.3%)	Akaraci et al. (2020); Gascon et al. (2015); Gascon et al. (2016); Houlden et al. (2018); Kondo et al. (2018); Lachowycz and Jones (2011); Luo et al. (2020); Shuvo et al. (2020); Twohig-Bennett and Jones (2018); van den Berg et al. (2015); Vanaken and Danckaerts (2018); Zhan et al. (2020)
Land-cover map	8 (22.2%)	de Keijzer et al. (2020); de Keijzer et al. (2016); Gascon et al. (2015); Gascon et al. (2016); Kabisch et al. (2017); Lambert et al. (2018); Twohig-Bennett and Jones (2018); Vanaken and Danckaerts (2018)
Duration of stay at greenspace	7 (19.4%)	Oh et al. (2017); Roberts et al. (2019); Shuvo et al. (2020); Vanaken and Danckaerts (2018); Whear et al. (2014); Yuan et al. (2021); Zhang et al. (2020)
Not clearly defined	7 (19.4%)	Carver et al. (2020); Gritzka et al. (2020); Shaffee and Abd Shukor (2018); Soga et al. (2017); Wen et al. (2019); Yeo et al. (2019); Zhang et al. (2017)
Quality of greenspace	6 (16.7%)	de Keijzer et al. (2016); Kondo et al. (2018); Shuvo et al. (2020); Twohig-Bennett and Jones (2018); van den Berg et al. (2015); Zhang et al. (2020)
Tree canopy	5 (13.9%)	Kabisch et al. (2017); Shuda et al. (2020); Twohig-Bennett and Jones (2018); van den Berg et al. (2015); Zhan et al. (2020)
Frequency to greenspace	4 (11.1%)	Shuvo et al. (2020); Twohig-Bennett and Jones (2018); Yuan et al. (2021); Zhang et al. (2020)
Other ^b^	19 (52.8%)	Akaraci et al. (2020); de Keijzer et al. (2020); de Keijzer et al. (2016); Dzhambov et al. (2014); Hartley et al. (2020); Houlden et al. (2018); Kabisch et al. (2017); Kondo et al. (2018); Lambert et al. (2017); Lambert et al. (2018); Luo et al. (2020); Schulz et al. (2018); Shuvo et al. (2020); Twohig-Bennett and Jones (2018); van den Berg et al. (2015); Vanaken and Danckaerts (2018); Yuan et al. (2021); Zhan et al. (2020); Zhang et al. (2020)

^a^ NDVI: normalised difference vegetation index.

^b^ ‘Other’: loss of trees from emerald ash bore disease, effect estimates for an increment of 10% all greenspace, percentage of greenspace, vegetation continuous fields, residential greenness, greenspace quantity, GPS and geographic simulation, built environment, aerial imagery, NDVI like-questionnaire, geolocation and timing, remote sensing data, satellite images, administrative data, spatial data, GIS data, street view images, different vegetation indices, tree counts through survey, land use classification/types, self-report, nature relatedness scale, access to a garden/greenspace, LiDAR imagery, street data density, number of parks in the area, pixel-based satellite imagery, detailed maps.

### Health outcomes

This review considered all health outcomes examined in relation to greenspace exposure. Given that over 110 health outcomes were identified ( Table 1), we categorised outcomes as: mental health and cognitive function, maternal health and birth outcomes, cardiovascular and metabolic outcomes, respiratory health and allergies, cancer, general health and quality of life (QoL), and all-cause and cause-specific mortality. Health outcomes which did not suit these categories were classified as other health outcomes ( Table 4).

**Table 4.** Health outcomes investigated in the included systematic reviews.

Health Outcome Category	Health Outcome	ICD-10 Code(s)	Review Articles
Mental health and cognitive function	Dementia	A50.4, B22.0, F00-F03, F84.3, G31.0	de Keijzer et al. (2016)
	Dementia-related behaviours	R45	Whear et al. (2014); Yeo et al. (2019)
	Overall mental health/psychological outcomes	F00-F99	Carver et al. (2020); de Keijzer et al. (2020); Gascon et al. (2015); Gritzka et al. (2020); Houlden et al. (2018); Islam et al. (2020); Kabisch et al. (2017); Kondo et al. (2018); Schulz et al. (2018); Shuvo et al. (2020); Soga et al. (2017); van den Berg et al. (2015); Vanaken and Danckaerts (2018); Wen et al. (2019); Zhang et al. (2017)
	Mood, affect	F06.3, F30-F39	Oh et al. (2017); Whear et al. (2014); Zhang et al. (2020)
	Depression	F32-F33	Carver et al. (2020); Oh et al. (2017); Roberts et al. (2019); Zhang et al. (2020)
	Stress	F43, Z73	Carver et al. (2020); Oh et al. (2017); Shaffee and Abd Shukor (2018); Shuda et al. (2020); Zhang et al. (2020)
	Behavioural and psychological distress, emotional wellbeing, neurocognitive development	F90-F98	de Keijzer et al. (2016); Gritzka et al. (2020); Islam et al. (2020); Kondo et al. (2018); Vanaken and Danckaerts (2018); Yeo et al. (2019); Zhang et al. (2020)
	Cognition	R40-R44	de Keijzer et al. (2020); de Keijzer et al. (2016)
Maternal health and birth outcomes	Birthweight	P07, P08	Akaraci et al. (2020); Dzhambov et al. (2014); Islam et al. (2020); Kabisch et al. (2017); Kondo et al. (2018); Lee et al. (2020); Zhan et al. (2020)
	Low birthweight	P07.0, P07.1	Akaraci et al. (2020); Lee et al. (2020); Zhan et al. (2020)
	Preterm birth	P07.2, P07.3	Akaraci et al. (2020); Islam et al. (2020); Kondo et al. (2018); Lee et al. (2020); Twohig-Bennett and Jones (2018)
	Gestational age	P07.2, P07.3, P08.2	Islam et al. (2020); Twohig-Bennett and Jones (2018); Zhan et al. (2020)
	Small for gestational age	P05.1	Akaraci et al. (2020); Dzhambov et al. (2014); Kondo et al. (2018); Twohig-Bennett and Jones (2018); Zhan et al. (2020)
	Pregnancy complications	O00-O99	Zhan et al. (2020)
	Head circumference	NA	Zhan et al. (2020)
Cardiovascular and metabolic outcomes	Cardiovascular health	I00-I99	Kondo et al. (2018)
	Diabetes	E10-E14, E23.2, O24	Kondo et al. (2018); Schulz et al. (2018); Twohig-Bennett and Jones (2018)
	Cholesterol	E78	de Keijzer et al. (2020); Twohig-Bennett and Jones (2018)
	Metabolic syndrome	E88.8	Lachowycz and Jones (2011)
	Hypertension	I10-I15	de Keijzer et al. (2020); Oh et al. (2017); Shaffee and Abd Shukor (2018); Shuda et al. (2020); Twohig-Bennett and Jones (2018)
	Stroke	I64	Twohig-Bennett and Jones (2018)
	Atherosclerosis	I70, I25.0	Oh et al. (2017)
	Hypotension	I95, O26.5	Oh et al. (2017); Shaffee and Abd Shukor (2018); Shuda et al. (2020); Twohig-Bennett and Jones (2018)
	Coronary heart disease	I25.9	Lachowycz and Jones (2011); Twohig-Bennett and Jones (2018)
	Heart rate	R00	Oh et al. (2017); Shaffee and Abd Shukor (2018); Shuda et al. (2020); Twohig-Bennett and Jones (2018)
Asthma, allergies, and respiratory health	Allergic diseases	D69.0, D72.1, H01.1, H65.1, H65.4, H65.9, J30, J45.0, J67.9, K52.2, L20.8, L23, L50.0, L56.1, M13.8, M30.1, T78.2, T78.4	Schulz et al. (2018)
	Acute respiratory illness	J00-J22	Schulz et al. (2018)
	Respiratory diseases	J00-J99	Kabisch et al. (2017); Kondo et al. (2018)
	Bronchitis	J20, J40-J42, J44, J45.0, J45.9	Islam et al. (2020)
	Allergic rhinitis	J30, J45.0	Lambert et al. (2017)
	Asthma	J44-J46, J82,	Hartley et al. (2020); Kabisch et al. (2017); Lambert et al. (2017); Twohig-Bennett and Jones (2018)
	Wheezing	R06.2	Islam et al. (2020)
	Allergic/atopic sensitisation	NA	Kabisch et al. (2017); Lambert et al. (2018)
Cancer	Cancer	C00-D48	Kabisch et al. (2017); Kondo et al. (2018); Schulz et al. (2018); Twohig-Bennett and Jones (2018)
	Lung cancer	C34	Twohig-Bennett and Jones (2018)
	Skin cancer	C43-C44	Twohig-Bennett and Jones (2018)
	Prostate cancer	C61	Kondo et al. (2018); Twohig-Bennett and Jones (2018)
General health and quality of life	Overall/general health/physiological outcomes	A00-Y98	Gascon et al. (2015); Schulz et al. (2018); Twohig-Bennett and Jones (2018); van den Berg et al. (2015); Wen et al. (2019); Yeo et al. (2019)
	Chronic diseases	A00-Q99	Islam et al. (2020)
	Overall physical health	E00-E90, G00-G99, I00-J99, M00-M99, R00-R99	de Keijzer et al. (2020); Shuvo et al. (2020); Soga et al. (2017); Yeo et al. (2019); Zhang et al. (2017)
	Sleep time and quality	NA	Whear et al. (2014)
	Medication use	NA	Whear et al. (2014)
	Quality of life	NA	Shuvo et al. (2020)
All-cause and cause specific mortality	All-cause mortality	A00-V98	Gascon et al. (2016); Kabisch et al. (2017); Rojas-Rueda et al. (2019); Twohig-Bennett and Jones (2018); van den Berg et al. (2015); Yuan et al. (2021)
	Cancer mortality	C00-D48	Twohig-Bennett and Jones (2018)
	Lung cancer mortality	C34	Gascon et al. (2016)
	Circulatory mortality	I00-I99	Lachowycz and Jones (2011)
	Ischaemic heart disease mortality	I20-I25	Yuan et al. (2021)
	Cardiovascular disease mortality	I51.6	Gascon et al. (2016); Schulz et al. (2018); Shuvo et al. (2020); Twohig-Bennett and Jones (2018); Yuan et al. (2021)
	Stroke mortality	I64	Yuan et al. (2021)
	Respiratory disease mortality	J00-J99	Yuan et al. (2021)
	Infant/neonatal mortality	P00-P04, P07-P08	Kabisch et al. (2017)
Other health outcomes	Cortisol level	E24, E27.1-E27.2, E27.8, E35.1	Kondo et al. (2018); Shaffee and Abd Shukor (2018); Shuda et al. (2020); Twohig-Bennett and Jones (2018)
	Atopic dermatitis	L20	Islam et al. (2020)
	Violence and agitation	R45	Kondo et al. (2018)
	Oxidative stress	NA	Oh et al. (2017)
	Immune markers	NA	Oh et al. (2017)
	Inflammation markers	NA	Oh et al. (2017)

ICD-10: International Classification of Diseases, Tenth Revision; NA: not available.

**Mental health and cognitive function.** Overall mental health was investigated in seven (19%) systematic reviews ( Carver et al., 2020; Gascon et al., 2015; Gritzka et al., 2020; Houlden et al., 2018; Shuvo et al., 2020; van den Berg et al., 2015; Zhang et al., 2017). van den Berg et al. (2015), Shuvo et al. (2020), Carver et al. (2020), and Gritzka et al. (2020) observed beneficial effects of greenspace exposure on overall mental health, but did not conduct meta-analyses due to heterogeneity in exposure and outcome measures. Similar findings were reported by Zhang et al. (2017), but the overall quality of original studies in the review was low and prone to a high risk of bias. In contrast, the relationship between surrounding greenness and overall mental health in children ( Gascon et al., 2015) and adults ( Gascon et al., 2015; Houlden et al., 2018) was inconclusive.

Impacts of greenspace exposure on psychological stress were also reported in seven (19%) systematic reviews ( de Keijzer et al., 2020; Gritzka et al., 2020; Kondo et al., 2018; Shaffee & Abd Shukor, 2018; Shuda et al., 2020; Soga et al., 2017; Zhang et al., 2020). Greenspace ( Kondo et al., 2018; Zhang et al., 2020) and nature ( Shaffee & Abd Shukor, 2018; Shuda et al., 2020) exposure, as well as participation in gardening activities ( Soga et al., 2017), were associated with reductions in stress. Greater long-term exposure to greenspace was also beneficial for stress ( de Keijzer et al., 2020). Gritzka et al. (2020) reported only one original study on the perceived stress of employees.

A total of 11 (31%) systematic reviews investigated mental health indicators ( Carver et al., 2020; Kondo et al., 2018; Oh et al., 2017; Roberts et al., 2019; Soga et al., 2017; Vanaken & Danckaerts, 2018; Wen et al., 2019; Yeo et al., 2019; Zhan et al., 2020; Zhang et al., 2020), although two of these reviews could not draw conclusions due to mixed results ( Kondo et al., 2018) and inadequate evidence ( Oh et al., 2017). Meta-analyses revealed that exposure to the natural environment was beneficial for depressive mood (SMD=0.38; 95%CI:0.16,0.56) ( Roberts et al., 2019) and that higher prenatal greenspace exposure was associated with lower odds of mental disorders (OR=0.87; 95%CI:0.77,0.99) ( Zhan et al., 2020). Other greenspace measures, such as gardening ( Soga et al., 2017), urban greenspace ( Kondo et al., 2018), long-term outdoor exposure ( de Keijzer et al., 2020), and indoor nature interventions ( Yeo et al., 2019) also had favourable effects on psychological wellbeing. In a narrative summary, Wen et al. (2019) found that negative emotions decreased after participating in forest bathing. Another review reported a positive association between mental health and greenspace exposure in older adults living in residential aged care facilities; however, health outcomes were primarily based on observations and perceptions of staff and relatives, which may be subject to information bias ( Carver et al., 2020). Greenspace exposure was also beneficially associated with emotional and behavioural difficulties in children ( Vanaken & Danckaerts, 2018) and psychological distress and depressive symptoms in adolescents ( Zhang et al., 2020).

Cognitive function was also explored in 10 (28%) reviews ( de Keijzer et al., 2020; de Keijzer et al., 2016; Gritzka et al., 2020; Islam et al., 2020; Kabisch et al., 2017; Kondo et al., 2018; Twohig-Bennett & Jones, 2018; Vanaken & Danckaerts, 2018; Whear et al., 2014; Yeo et al., 2019). Greenspace exposure was associated with improved cognitive function and development in adults ( Gritzka et al., 2020; Kondo et al., 2018) and children ( Islam et al., 2020; Kondo et al., 2018). Garden use was also associated with decreased agitation in people with dementia ( Whear et al., 2014). Other reviews concluded that evidence on cognition was limited ( de Keijzer et al., 2020; de Keijzer et al., 2016; Vanaken & Danckaerts, 2018), inconsistent ( Yeo et al., 2019), or insufficient ( Kabisch et al., 2017; Twohig-Bennett & Jones, 2018).

**Maternal health and birth outcomes.** The impact of greenspace exposure on pregnancy outcomes such as birthweight (BW), low birth weight (LBW), small for gestational age (SGA), and preterm birth (PTB), was assessed in eight (22%) systematic reviews ( Akaraci et al., 2020; Dzhambov et al., 2014; Islam et al., 2020; Kabisch et al., 2017; Kondo et al., 2018; Lee et al., 2020; Twohig-Bennett & Jones, 2018; Zhan et al., 2020). Kondo et al. (2018) found insufficient evidence to support a statistical association between greenspace exposure and BW, PTB, and SGA, while Kabisch et al. (2017) reported inconsistent evidence on BW. All other reviews found weak but positive associations between higher surrounding greenness and increased BW ( Akaraci et al., 2020; Dzhambov et al., 2014; Islam et al., 2020), decreased risk for LBW ( Islam et al., 2020) and PTB ( Twohig-Bennett & Jones, 2018), and lower odds of SGA ( Akaraci et al., 2020) within different greenspace buffer sizes. For example, a meta-analysis of ten studies by Zhan et al. (2020) demonstrated protective effects of residential greenness on LBW measured by NDVI at 100 (OR=0.80; 95%CI:0.75,0.99), 300 (OR=0.82; 95%CI:0.71,0.93), and 500 (OR=0.85; 95%CI:0.77,0.93) metre buffers. Lee et al. (2020) also found that BW was associated with overall greenness. The association was determined via NDVI buffer sizes of 100 (β [beta standardised regression coefficient] **=**0.003; 95%CI:0.001,0.005), 250 (β=0.001; 95%CI:0.000,0.002), and 500 (β=0.002; 95%CI:0.001,0.004) metres after adjusting for air quality and civil environment.

**Cardiovascular and metabolic outcomes.** The risk of cardiovascular outcomes following greenspace exposure was assessed in ten (28%) systematic reviews ( de Keijzer et al., 2020; Gritzka et al., 2020; Kabisch et al., 2017; Kondo et al., 2018; Lachowycz & Jones, 2011; Oh et al., 2017; Twohig-Bennett & Jones, 2018; Wen et al., 2019; Yeo et al., 2019; Yuan et al., 2021). A review of observational studies by Yuan et al. (2021) reported beneficial effects of greenspace on the risk of cardiovascular disease (CVD) events in older adult populations. The authors did not conduct a meta-analysis due to the small number of original studies on CVD events. Wen et al. (2019) reported an improvement in overall cardiovascular function and hemodynamic index in response to forest bathing. Limited evidence was found on coronary heart disease (CHD) ( Lachowycz & Jones, 2011) and hypertension ( de Keijzer et al., 2020; Twohig-Bennett & Jones, 2018). Gritzka et al. (2020) reported inconclusive findings on blood pressure (BP), while other reviews found only one ( Kabisch et al., 2017; Kondo et al., 2018; Yeo et al., 2019) or two ( Oh et al., 2017) original studies on the outcome. However, the meta-analyses by Twohig-Bennett and Jones (2018) demonstrated decreased diastolic blood pressure (DBP) (MD=-1.97; 95%CI:-3.45,-0.49), heart rate (HR) (MD=-2.57; 95%CI:-4.30,-0.83), and heart rate variability (HRV) (MD=91.87; 95%CI:50.92,132.82) following exposure to increased greenery.

**Respiratory health and allergies.** We identified nine (25%) systematic reviews that assessed exposure to residential greenspace on respiratory health ( de Keijzer et al., 2020; Hartley et al., 2020; Islam et al., 2020; Kabisch et al., 2017; Kondo et al., 2018; Lambert et al., 2017, 2018; Oh et al., 2017; Twohig-Bennett & Jones, 2018). Lambert et al. (2017) demonstrated no association between residential greenness and allergic respiratory diseases in children and adolescents. Hartley et al. (2020) undertook an updated review of Lambert et al. (2017) and found similar results. Meta-analysis showed that high greenspace areas were associated with lower odds of asthma, although this result was based on only two original studies with substantial heterogeneity ( Twohig-Bennett & Jones, 2018). Islam et al. (2020) and Lambert et al. (2018) investigated the respiratory health of children and adolescents, which revealed limited evidence to support the effects of greenspace exposure on asthma and atopic sensitisation, respectively. Kabisch et al. (2017), Kondo et al. (2018), de Keijzer et al. (2020), and Oh et al. (2017) reported insufficient original studies to draw conclusions on respiratory diseases, pulmonary function, and aeroallergens.

**Cancer.** Only four (11%) systematic reviews investigated the relationship between greenspace exposure and cancer ( de Keijzer et al., 2020; Kabisch et al., 2017; Kondo et al., 2018; Twohig-Bennett & Jones, 2018) due to a small number of original studies on the outcome. Kondo et al. (2018) and Twohig-Bennett and Jones (2018) suggested that greenspace exposure is beneficially associated with the risk of prostate cancer after controlling for individual factors, including PA, smoking, and medical history. de Keijzer et al. (2020) and Twohig-Bennett and Jones (2018) also suggested a harmful association between greenspace exposure and skin cancer. Inconclusive findings were reported for the effect of park availability on lung cancer ( Kabisch et al., 2017).

**General health and Quality of life (QoL).** We found 11 (31%) systematic reviews that investigated the role of greenspace exposure on self-reported general health, perceived wellbeing, and QoL ( de Keijzer et al., 2020; Gritzka et al., 2020; Houlden et al., 2018; Kondo et al., 2018; Shuvo et al., 2020; Soga et al., 2017; Twohig-Bennett & Jones, 2018; van den Berg et al., 2015; Vanaken & Danckaerts, 2018; Whear et al., 2014; Yeo et al., 2019). Some reviews reported inconclusive findings on the effect of greenspace exposure on perceived ( de Keijzer et al., 2020; Gritzka et al., 2020) and objective ( Houlden et al., 2018) wellbeing and QoL, while another found a positive association ( Vanaken & Danckaerts, 2018). Shuvo et al. (2020) concluded that there is an association between greenspace exposure and self-reported general health, but the methodological quality of studies was low. Earlier reviews revealed mixed evidence on the relationship between greenness and general health ( Kondo et al., 2018; van den Berg et al., 2015) and falls ( Whear et al., 2014). A meta-analysis by Twohig-Bennett and Jones (2018) demonstrated an association between greenspace exposure and self-reported health conditions (OR=1.12; 95%CI:1.05,1.19). Another two reviews considered greenspace exposure via participation in gardening activities and reported beneficial effects on self-perceived health and QoL ( Soga et al., 2017; Yeo et al., 2019).

**All-cause and cause-specific mortality.** A total of 11 (31%) systematic reviews and meta-analyses considered all-cause or cause-specific mortality ( Akaraci et al., 2020; Gascon et al., 2016; Islam et al., 2020; Kabisch et al., 2017; Kondo et al., 2018; Lachowycz & Jones, 2011; Rojas-Rueda et al., 2019; Twohig-Bennett & Jones, 2018; van den Berg et al., 2015; Yuan et al., 2021; Zhan et al., 2020). The meta-analysis by Gascon et al. (2016) found that living in areas of high greenery reduced the risk of all-cause mortality (RR=0.92; 95%CI:0.87,0.97) but not lung cancer mortality. Three meta-analyses performed after Gascon et al. (2016) presented similar findings ( Rojas-Rueda et al., 2019; Twohig-Bennett & Jones, 2018; Yuan et al., 2021), except for CVD mortality ( Yuan et al., 2021). Yuan et al. (2021) also reported that increased NDVI is associated with reduced risk of stroke mortality (HR=0.77; 95%CI:0.59,1.00). Narrative syntheses summarised moderate to strong effects of increased greenness on the risk of the following mortalities: non-accidental ( van den Berg et al., 2015), CVD ( Kondo et al., 2018), respiratory ( Kondo et al., 2018), heat-wave ( Kabisch et al., 2017) and overall cancer ( Kondo et al., 2018; Twohig-Bennett & Jones, 2018). Insufficient evidence was found on neonatal ( Akaraci et al., 2020), infant ( Islam et al., 2020; Zhan et al., 2020), and circulatory disease ( Lachowycz & Jones, 2011) mortality for synthesis.

**Other health outcomes.** We identified 16 (44%) systematic reviews that evaluated the effect of greenspace on other health outcomes. Inconsistent associations ( Kabisch et al., 2017; Kondo et al., 2018; Lachowycz & Jones, 2011; Yeo et al., 2019) or no association ( Gritzka et al., 2020) were reported for greenspace exposure and obesity-related health indicators. In contrast, a meta-analysis by Luo et al. (2020) reported higher NDVI to be associated with lower odds of being overweight or obese (OR=0.88; 95%CI:0.84,0.91). Other reviews found inadequate evidence on weight status ( de Keijzer et al., 2020; Schulz et al., 2018; Shuvo et al., 2020). Kondo et al. (2018) reported mixed results on the association between urban greenspace exposure and cortisol concentration, while other reviews found insufficient evidence on serum ( Gritzka et al., 2020; Oh et al., 2017) and salivary ( Shuda et al., 2020) cortisol. In comparison, a meta-analysis by Twohig-Bennett and Jones (2018) showed that salivary cortisol was lower (MD=-0.05; 95%CI:-0.07,-0.04) when exposed to higher levels of greenspace. Narrative synthesis by Wen et al. (2019) also revealed a decrease in cortisol and adrenaline in response to forest bathing.

### Quantitative data summary

Quantitative data collected from meta-analyses were collated according to outcome category, outcome, and measure of greenspace exposure ( Table 5). Effect measures reported in meta-analyses which were based on a singular original study were excluded from the quantitative data summary table. No meta-analyses were reported for cancer outcomes.

**Table 5.** Listing of quantitative data from included reviews’ meta-analyses.

Outcome	Review Article	No. of original studies included	Exposure measure	Effect measure	Estimate (95%CI) from meta-analysis
Mental health and cognitive function					
Depressive mood	Roberts et al. (2019)	30	Short-term exposure to natural environment	SMD	0.05 (0.04,0.05)
Depressive mood	Roberts et al. (2019)	3	Short-term exposure to natural environment	SMD	0.38 (0.16,0.56)
Maternal health and birth outcomes					
Birthweight	Akaraci et al. (2020)	20	NDVI increase within 250/300m	Standardised regression coefficient	0.001 (0.0002,0.002)
Birthweight	Zhan et al. (2020)	17	NDVI within 100m	Standardised regression coefficient	20.22 (13.50,26.93)
Birthweight	Zhan et al. (2020)	14	NDVI within 250m	Standardised regression coefficient	11.08 (6.79,15.37)
Birthweight	Zhan et al. (2020)	4	NDVI within 300m	Standardised regression coefficient	23.15 (7.60,38.69)
Birthweight	Zhan et al. (2020)	13	NDVI within 500m	Standardised regression coefficient	22.41 (11.01,33.82)
Birthweight	Zhan et al. (2020)	7	NDVI within 1000m	Standardised regression coefficient	16.72 (0.84,32.60)
Birthweight	Zhan et al. (2020)	2	Percentage tree canopy within 300m	Standardised regression coefficient	2.28 (0.08,4.48)
Birthweight	Zhan et al. (2020)	2	Percentage tree canopy within >300m	Standardised regression coefficient	-3.47 (-7.44,0.50)
Birthweight	Dzhambov et al. (2014)	7	Redidential greenness within 100m	r (correlation)	0.05 (0.04,0.06)
Birthweight	Lee et al. (2020)	4	NDVI	Standardised regression coefficient	0.001 (0.001,0.002)
Birthweight	Lee et al. (2020)	6	NDVI within 100m	Standardised regression coefficient	0.003 (0.002,0.004)
Small for gestational age	Akaraci et al. (2020)	14	NDVI increase within 250/300m	Pooled OR	0.95 (0.92,0.97)
Small for gestational age	Zhan et al. (2020)	6	NDVI within 100m	OR	0.93 (0.88,1.00)
Small for gestational age	Zhan et al. (2020)	6	NDVI within 250m	OR	0.95 (0.88,1.03)
Small for gestational age	Zhan et al. (2020)	4	NDVI within 300m	OR	0.85 (0.67,1.09)
Small for gestational age	Zhan et al. (2020)	6	NDVI within 500m	OR	0.90 (0.73,1.12)
Small for gestational age	Zhan et al. (2020)	3	NDVI within 1000m	OR	0.83 (0.60,1.16)
Small for gestational age	Twohig-Bennett and Jones (2018)	4	High vs low * greenspace area	MD	0.81 (0.76,0.86)
Low birth weight	Akaraci et al. (2020)	11	NDVI increase within 250/300m	Pooled OR	0.96 (0.91,1.01)
Low birth weight	Zhan et al. (2020)	7	NDVI within 100m	OR	0.86 (0.75,0.99)
Low birth weight	Zhan et al. (2020)	4	NDVI within 250m	OR	0.90 (0.81,1.00)
Low birth weight	Zhan et al. (2020)	5	NDVI within 300m	OR	0.82 (0.71,0.93)
Low birth weight	Zhan et al. (2020)	8	NDVI within 500m	OR	0.85 (0.77,0.93)
Low birth weight	Zhan et al. (2020)	3	NDVI within 1000m	OR	0.77 (0.48,1.24)
Low birth weight	Zhan et al. (2020)	2	Distance to nearest greenspace	OR	1.10 (0.97,1.25)
Low birth weight	Lee et al. (2020)	5	NDVI	OR	0.94 (0.92,0.97)
Preterm birth	Akaraci et al. (2020)	11	NDVI increase within 250/300m	Pooled OR	0.99 (0.97,1.02)
Preterm birth	Lee et al. (2020)	5	NDVI	OR	0.98 (0.97,0.99)
Preterm birth	Twohig-Bennett and Jones (2018)	6	High vs low greenspace area	MD	0.87 (0.80,0.94)
Preterm birth	Zhan et al. (2020)	3	Percentage greenspace within >300m	OR	1.02 (0.93,1.11)
Gestational age and head circumference	Zhan et al. (2020)	5	NDVI within >300m	Standardised regression coefficient	1.73 (0.69,2.76)
Gestational age	Twohig-Bennett and Jones (2018)	3	High vs low greenspace exposure	MD	<-0.01 (-0.05,0.05)
Gestational diabetes	Zhan et al. (2020)	2	NDVI within 300m	OR	0.81 (0.57,1.15)
Mental health (as a pregnancy complication)	Zhan et al. (2020)	2	NDVI within >300m	OR	0.87 (0.77,0.99)
Cardiovascular and metabolic outcomes					
Heart rate	Twohig-Bennett and Jones (2018)	10	High vs low greenspace exposure	MD	-2.57 (-4.30,-0.83)
Hypertension	Twohig-Bennett and Jones (2018)	4	High vs low greenspace area	MD	0.99 (0.81,1.20)
Stroke	Twohig-Bennett and Jones (2018)	3	High vs low greenspace area	MD	0.82 (0.61,1.11)
Coronary heart disease	Twohig-Bennett and Jones (2018)	2	High vs low greenspace area	MD	0.92 (0.78,1.07)
HDL cholesterol	Twohig-Bennett and Jones (2018)	2	High vs low greenspace exposure	MD	-0.03 (-0.05,0.00)
LDL cholesterol	Twohig-Bennett and Jones (2018)	2	High vs low greenspace exposure	MD	0.04 (-0.03,0.11)
Total cholesterol	Twohig-Bennett and Jones (2018)	2	High vs low greenspace exposure	MD	0.03 (-0.05,0.10)
Diastolic blood pressure	Twohig-Bennett and Jones (2018)	12	High vs low greenspace exposure	MD	-1.97 (-3.45,-0.49)
Systolic blood pressure	Twohig-Bennett and Jones (2018)	13	High vs low greenspace exposure	MD	-1.50 (-3.43,0.44)
High frequency power of heart rate variability	Twohig-Bennett and Jones (2018)	7	High vs low greenspace exposure	MD	91.87 (50.92,132.82)
Low frequency heart rate variability	Twohig-Bennett and Jones (2018)	6	High vs low greenspace exposure	MD	-0.06 (-0.08,-0.03)
HbA1c	Twohig-Bennett and Jones (2018)	2	High vs low greenspace exposure	MD	-0.77 (-1.86,0.32)
Fasting blood glucose	Twohig-Bennett and Jones (2018)	2	High vs low greenspace exposure	MD	-0.01 (-0.08,0.07)
Type 2 diabetes	Twohig-Bennett and Jones (2018)	6	High vs low greenspace area	MD	0.72 (0.61,0.85)
Triglycerides	Twohig-Bennett and Jones (2018)	2	High vs low greenspace exposure	MD	0.06 (-0.01,0.12)
Dyslipidaemia	Twohig-Bennett and Jones (2018)	2	High vs low greenspace area	MD	0.94 (0.75,1.17)
Respiratory health and allergies					
Asthma	Lambert et al. (2017)	4	NDVI within 100m	OR	1.01 (0.93,1.09)
Asthma	Twohig-Bennett and Jones (2018)	2	High vs low greenspace area	MD	0.93 (0.57,1.52)
Allergic rhinitis	Lambert et al. (2017)	6	NDVI within 500m	OR	0.99 (0.87,1.12)
Atopic sensitisation	Lambert et al. (2018)	6	NDVI within 500m	OR	0.96 (0.75,1.22)
Atopic sensitisation	Lambert et al. (2018)	4	NDVI within 500m	OR	0.85 (0.61,1.18)
General health and quality of life					
Overweight/obesity	Luo et al. (2020)	4	Proximity to nearest greenspace	OR	0.99 (0.99,1.00)
Overweight/obesity	Luo et al. (2020)	6	NDVI	OR	0.88 (0.84,0.91)
Overweight/obesity	Luo et al. (2020)	4	Number of parks	OR	0.98 (0.97,1.00)
Overweight/obesity	Luo et al. (2020)	6	Proportion of greenspace	OR	0.96 (0.85,1.08)
Any health outcome	Soga et al. (2017)	21	Gardening vs control	SMD	0.42 (0.36,0.48)
Good self-reported health	Twohig-Bennett and Jones (2018)	10	High vs low greenspace area	MD	1.12 (1.05,1.19)
All-cause and cause-specific mortality					
All-cause mortality	Yuan et al. (2021)	8	Per 0.1 unit of NDVI	Pooled HR	0.99 (0.97,1.00)
All-cause mortality	Rojas-Rueda et al. (2019)	9	Per 0.1 unit of NDVI within <500m	Pooled HR	0.96 (0.94,0.97)
All-cause mortality	Gascon et al. (2016)	6	Per 10% increase in NDVI	RR	0.92 (0.87,0.97)
All-cause mortality	Twohig-Bennett and Jones (2018)	4	High vs low greenspace area	MD	0.69 (0.55,0.87)
CVD mortality	Yuan et al. (2021)	8	Per 0.1 unit of NDVI	Pooled HR	0.99 (0.89,1.09)
CVD mortality	Gascon et al. (2016)	8	High vs low greenspace exposure	RR	0.96 (0.94,0.97)
Cardiovascular mortality	Twohig-Bennett and Jones (2018)	2	High vs low greenspace area	MD	0.84 (0.76,0.93)
Ischaemic heart disease mortality	Yuan et al. (2021)	8	Per 0.1 unit of NDVI	Pooled HR	0.96 (0.88,1.05)
Respiratory disease mortality	Yuan et al. (2021)	8	Per 0.1 unit of NDVI	Pooled HR	0.99 (0.89,1.10)
Lung cancer mortality	Gascon et al. (2016)	4	High vs low greenspace exposure	RR	0.98 (0.95,1.02)
Stroke mortality	Yuan et al. (2021)	8	Per 0.1 unit of NDVI	Pooled HR	0.77 (0.59,1.00)
Other health outcomes					
Salivary cortisol	Twohig-Bennett and Jones (2018)	7	NDVI	MD	-0.05 (-0.07,-0.04)

SMD: standardised mean difference; NDVI: normalised difference vegetation index; OR: odds ratio; MD: mean difference; LDL: low density lipoprotein; HDL: high density lipoprotein; HR: hazard ratio; RR: risk ratio; CVD: cardiovascular disease.

* High vs low greenspace exposure or greenspace area was determined for each greenspace measure, which was then used to calculate the effect estimate for each health outcome. Please refer to the relevant systematic review for further information on the greenspace measures used.

### Quality of Evidence and the risk of bias

**Figure 1.**
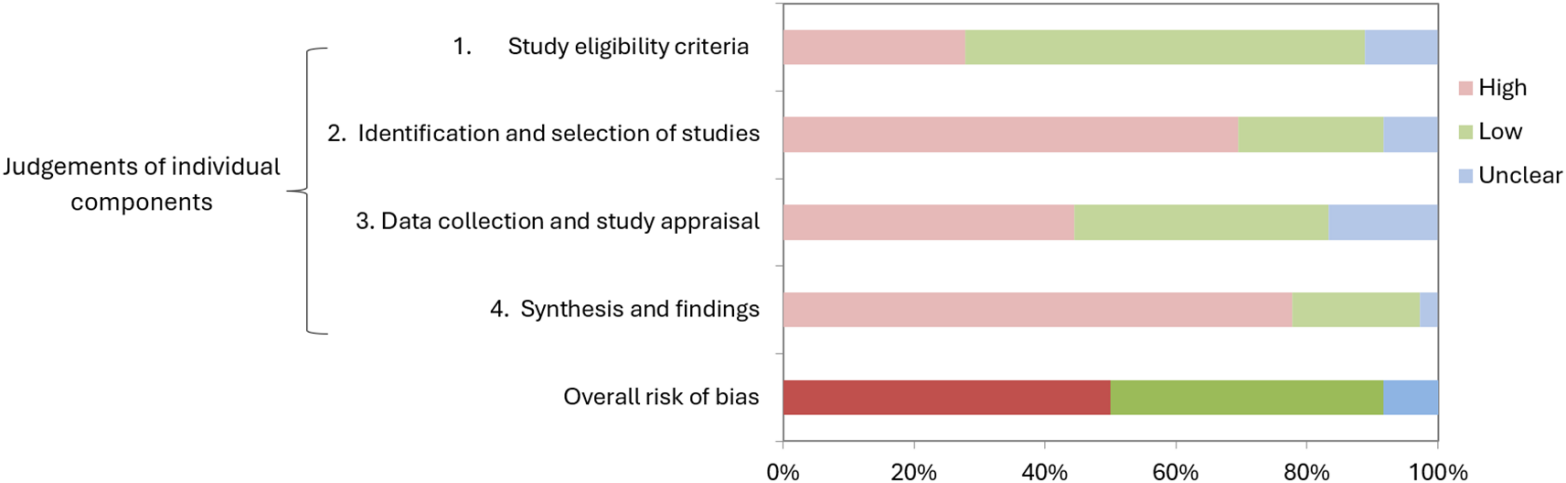
Risk of bias assessment of included reviews using ROBIS. Rows 1-4 show judgements on individual criteria while the bottom row shows the overall risk of bias.

Half of the systematic reviews (50%) had a high risk of bias, and three (8%) reviews were categorised as having ‘unclear risk’. The remaining 15 (42%) reviews were deemed low risk using the ROBIS tool ( Figure 1; extended data: Supplementary Table S2).

Of the 36 systematic reviews, eight (22%) were rated as high quality, 15 (42%) as moderate quality, and 13 (36%) as low quality according to the AMSTAR-2 tool ( Table 6; extended data: Supplementary Figure S1). No reviews were rated as critically low-quality.

**Table 6.** Methodological quality assessment of included systematic reviews using AMSTAR-2.

Question number ^a^	1	2	3	4	5	6	7	8	9	10	11	12	13	14	15	16	Quality
Author, Year																	
Akaraci et al. (2020)	N ^b^	PY	N	PY	Y	Y	N	PY	NA, PY	N	NA, Y	Y	N	Y	Y	Y	L
Carver et al. (2020)	Y	PY	N	PY	Y	N	N	PY	NA, N	N	NA, N	N	N	N	N	Y	L
de Keijzer et al. (2020)	N	PY	N	PY	Y	N	N	PY	NA, N	N	NA, N	N	N	Y	N	Y	H
de Keijzer et al. (2016)	N	PY	N	PY	N	N	PY	PY	NA, N	N	NA, N	N	N	PY	N	Y	H
Dzhambov et al. (2014)	Y	PY	N	Y	Y	Y	Y	PY	NA, Y	N	NA, PY	PY	Y	Y	Y	N	M
Gascon et al. (2016)	Y	PY	N	PY	Y	Y	PN	PY	NA, PY	N	NA, PY	PY	N	PY	Y	N	L
Gascon et al. (2015)	Y	PY	N	Y	Y	Y	Y	N	NA, PY	N	N*, N*	N*	PY	PY	N*	Y	H
Gritzka et al. (2020)	Y	PY	N	PY	Y	Y	Y	PY	NA, PY	N	N*, N*	N*	Y	Y	N*	Y	M
Hartley et al. (2020)	Y	PY	N	PY	Y	N	N	PY	NA, N	N	NA, N	N	N	PY	N	Y	L
Houlden et al. (2018)	Y	PY	N	PY	Y	N	Y	PY	NA, PY	N	N*, N*	N*	Y	PY	N*	Y	M
Islam et al. (2020)	N	N	N	PY	N	N	Y	N	NA, N	N	N*, N*	N*	N	N	N*	Y	L
Kabisch et al. (2017)	Y	N	N	PY	N	N	N	N	NA, N	N	N*, N*	N*	Y	N	N*	Y	L
Kondo et al. (2018)	N	N	Y	PY	N	N	Y	PY	NA, N	N	N*, N*	N*	N	N	N*	Y	L
Lachowycz and Jones (2011)	N	PY	N	PY	Y	N	Y	PY	NA, N	N	N*, N*	N*	N	PY	N*	Y	L
Lambert et al. (2018)	Y	Y	N	PY	Y	Y	Y	PY	NA, Y	N	N*, N*	N*	Y	Y	N*	Y	M
Lambert et al. (2017)	Y	Y	N	PY	Y	Y	Y	PY	NA, Y	N	NA, Y	Y	Y	Y	N	Y	L
Lee et al. (2020)	Y	PY	N	PY	N	N	Y	PY	NA, N	N	NA, PY	Y	N	N	Y	N	H
Luo et al. (2020)	N	Y	N	PY	Y	Y	Y	PY	NA, PY	N	NA, Y	Y	Y	Y	Y	Y	M
Oh et al. (2017)	Y	PY	N	PY	Y	Y	PY	PY	NA, Y	N	N*, N*	N*	Y	N	N*	Y	M
Roberts et al. (2019)	Y	Y	N	PY	Y	Y	Y	Y	NA, Y	N	NA, Y	Y	Y	Y	Y	Y	H
Rojas-Rueda et al. (2019)	N	PY	N	PY	Y	Y	Y	PY	NA, PY	N	NA, Y	Y	Y	Y	Y	Y	H
Schulz et al. (2018)	Y	PY	N	PY	N	N	Y	PY	NA, N	N	N*, N*	N*	Y	Y	N*	Y	H
Shaffee and Abd Shukor (2018)	N	N	N	PY	N	N	N	PY	NA, N	N	N*, N*	N*	N	N	N*	Y	L
Shuda et al. (2020)	Y	PY	N	PY	N	N	PY	PY	NA, PY	N	N*, N*	N*	Y	N	N*	N	M
Shuvo et al. (2020)	N	PY	N	PY	Y	N	Y	N	NA, PY	N	N*, N*	N*	Y	Y	N*	Y	M
Soga et al. (2017)	Y	PY	N	PY	N	N	Y	PY	NA, N	N	NA, Y	Y	N	Y	Y	Y	L
Twohig-Bennett and Jones (2018)	Y	PY	N	PY	Y	Y	Y	PY	NA, PY	N	NA, Y	Y	PY	Y	Y	Y	M
Vanaken and Danckaerts (2018)	Y	N	N	PY	N	N	N	Y	NA, N	N	N*, N*	N*	N	Y	N*	Y	L
van den Berg et al. (2015)	N	PY	N	PY	Y	Y	Y	PY	NA, PY	N	N*, N*	N*	PY	N	N*	Y	M
Wen et al. (2019)	N	PY	N	PY	N	Y	Y	N	NA, Y	N	N*, N*	N*	Y	N	N*	Y	M
Whear et al. (2014)	Y	PY	N	Y	Y	N	Y	Y	NA, Y	N	N*, N*	N*	Y	N	N*	Y	M
Yeo et al. (2019)	Y	PY	N	Y	Y	N	Y	Y	NA, PY	N	N*, N*	N*	Y	N	N*	Y	M
Yuan et al. (2021)	Y	PY	N	PY	Y	Y	Y	PY	NA, Y	N	NA, Y	Y	Y	Y	Y	Y	M
Zhan et al. (2020)	Y	PY	N	PY	Y	Y	Y	PY	NA, PY	N	NA, Y	Y	Y	Y	Y	Y	M
Zhang et al. (2020)	Y	N	Y	PY	N	N	PY	PY	NA, N	N	N*, N*	N*	Y	Y	N*	Y	H
Zhang et al. (2017)	Y	PY	N	PY	N	N	Y	PY	NA, N	N	N*, N*	N*	Y	N	N*	Y	L

^a^ 1: Participant, Intervention, Comparison, Outcome (PICO) components, 2: Pre-established protocol, 3: Explanation of included studies’ design, 4: Comprehensive search strategy, 5: Study selection in duplicate, 6: Data extraction in duplicate, 7: List of excluded studies with justification, 8: Description of included studies, 9: Assessment of risk of bias (RoB) in included studies, 10: Funding sources of included studies, 11: Use of appropriate statistical methods, 12: RoB impact on synthesised results, 13: Results interpretation with RoB reference, 14: Heterogeneity explanation, 15: Publication/small study bias investigation, 16: Conflict of interest declaration.

^b^ Y = Yes; P/Y= Partial Yes; N = No; NA = Not applicable (no meta-analysis conducted); CL = Critically low; L = Low; M = Moderate; H = High.

## Discussion

This umbrella review summarises 36 systematic reviews on the relationship between greenspace exposure and health outcomes. Our study yielded consistent results with existing umbrella reviews for the beneficial effects of greenspace exposure on mental health and cognitive function ( Stier-Jarmer et al., 2021; Yang et al., 2021; Zare Sakhvidi et al., 2022), non-accidental/all-cause mortality ( van den Bosch & Ode Sang, 2017; Yang et al., 2021), and CVD-mortality ( Yang et al., 2021). Additionally, we observed positive associations between greenspace exposure and maternal health and birth outcomes, including PTB and SGA which were not present in other reviews ( Yang et al., 2021). However, these associations were less consistent than those for mental health and cognitive function. Stier-Jarmer et al. (2021) and Yang et al. (2021) also reported beneficial effects of greenspace on cardiovascular health and cardiometabolic factors, respectively, which were not replicated in our study. This may be attributable to different inclusion and exclusion criteria between umbrella reviews. Finally, we identified mixed, limited, or no association between greenspace exposure and respiratory health and allergies, general health and QoL, cancer, and other health outcomes, which is congruous with other umbrella reviews ( Yang et al., 2021; Zare Sakhvidi et al., 2022). Overall, our review suggests that there may be an association between greenspace exposure and human health and wellbeing.

The variety of greenspace measures used may contribute to the ambivalent findings reported. Some studies used objective measures such as NDVI, proximity to greenspace, and land cover maps to quantify greenspace exposure whereas others used subjective measures including street view images and self-report questionnaires ( Table 3). This precluded many systematic reviews from conducting meta-analyses which restricted the number of pooled estimates available for synthesis in this review.

Reduced access to natural spaces and biodiversity has limited the population’s access to the physical, mental, and cognitive health benefits of these spaces ( Puplampu & Boafo, 2021). Urbanisation has increased in response to a growing urban population and has led to fragmentation and declines in natural ecosystems ( Kingsley, 2019; Li et al., 2019). As a result, homes located near greenspaces have become more expensive, increasing inequalities in access to greenspaces based on socioeconomic status (SES) ( Sharifi et al., 2021). Studies have also shown that most indoor workers do not go outside during office hours, further reducing access to greenspace and the associated health benefits ( Gilchrist et al., 2015; Lottrup et al., 2013). These restrictions may have influenced the relationship between greenspace exposure and health outcomes in the included systematic reviews.

A variety of pathways, mediators, and effect modifiers have been suggested for the relationship between greenspace exposure and health outcomes, including comorbidities, genetics, and PA. Comorbidities, which typically exacerbate other health conditions, may attenuate the effect of greenspace exposure on the primary health outcome ( Godina et al., 2023; Walsan et al., 2020). Genetic variation has also been suggested as an effect modifier of the relationship between greenspace and health outcomes, but initial studies have reported insignificant findings ( Cohen-Cline et al., 2015; Engemann et al., 2020). Conversely, PA and social interaction, which are beneficial for many health conditions, have been suggested as mediators of the relationship between greenspace exposure and health outcomes ( Zhang et al., 2022). Greenspace exposure may improve mental, physical, and cognitive health by encouraging participation in outdoor PA ( Sun et al., 2022; van den Berg et al., 2019) and social interaction ( Dadvand et al., 2019; Orstad et al., 2020). Additional investigation into the exact role of comorbidities, genetics, and PA, as well as other effect modifiers such as age, sex, SES, race, and urbanicity is required.

Interactions between greenspace and other environmental factors including air pollution, chemical exposures, toxins, and smoking may also influence the effect of greenspace on health outcomes. Environmental contaminants are recognised as detrimental to human health with the ability to induce and/or exacerbate many health conditions ( Brusseau et al., 2019). Natural greenery can minimise the harmful effects of these environmental issues via air purification, as well as the prevention of soil contamination and erosion ( Qiu et al., 2021; Yang et al., 2015). Reduction of these environmental contaminants by green ecosystems can lead to improvements in health conditions, particularly respiratory symptoms and CVD ( Boelee et al., 2019; Schraufnagel et al., 2019).

We conducted a thorough review of systematic reviews and meta-analyses on the impact of greenspace exposure on health outcomes. In addition to the wide range of databases searched, a manual reference search was conducted to ensure all relevant articles were identified. All stages of screening and data collection, as well as risk of bias and methodological quality assessment were conducted independently by two authors. We outlined the types and frequency of greenspace measures used and allocated ICD-10 codes to health outcomes investigated in each systematic review. For each health outcome reported in meta-analyses, we provided the measure of greenspace and the numerical estimate of the association, as well as the number of original articles this estimate is based on. These methods allowed for a more holistic overview of the current literature and should be considered for use in future umbrella reviews.

Despite the contributions of this umbrella review, several limitations highlight the need for further research to strengthen the evidence on greenspace exposure and health outcomes. Current evidence, including this umbrella review, has been unable to establish a causal link between greenspace exposure and health outcomes, as most studies are observational. A small number of RCTs in our review, along with inconsistencies in greenspace and effect measures, pose challenges in drawing definitive conclusions on the greenspace-health association.

Current evidence on potential interactions, mediators, and effect modifiers of this relationship is also limited and conflicting, and further research is needed to assess the influence of genetics and environmental exposures ( Zhang et al., 2022). To account for new evidence, we conducted an update of this umbrella review ( Bryer et al., 2022).

## Conclusion

This umbrella review synthesised systematic reviews and meta-analyses on the effect of greenspace exposure on health outcomes. Beneficial effects were found for all-cause and CVD mortality, mental health and cognitive function, and maternal health and birth outcomes. Ambivalent results were found for cardiovascular and metabolic health, respiratory health and allergies, and general health and QOL. There were limited systematic reviews available for assessing cancer outcomes. In light of these diverse findings, it is apparent that the current evidence on the relationship between greenspace exposure and health outcomes is characterised by inconsistencies. Nevertheless, this umbrella review highlights the association between greenspace and a variety of health outcomes.

## Data Availability

No data are associated with this article. Open Science Framework (OSF): Greenspace exposure and associated health outcomes: a systematic review of reviews. Supplementary Material. DOI 10.17605/OSF.IO/U39EK ( Bryer et al., 2024). This project contains the following extended data:
• Supplementary Table S1. (Literature search strategies) • Supplementary Table S2. (Risk of bias assessment by domain) • Supplementary Figure S1: (Overall methodological quality) • Supplementary File S1. (Systematic reviews excluded during full-text screening) Open Science Framework (OSF): PRISMA checklist and flow chart for Greenspace exposure and associated health outcomes: a systematic review of reviews. DOI 10.17605/OSF.IO/U39EK ( Bryer et al., 2024). Data are available under the terms of the Creative Commons Attribution 4.0 International license (CC-BY 4.0).

## Software availability

Endnote version 20 was used as a reference management tool in this study ( The EndNote Team, 2013). A free alternative to Endnote is Zotero ( Zotero, 2024).

Covidence was used to streamline eligibility screening and data extraction in this study ( Covidence systematic review software, 2022). A free alternative to Covidence in Rayyan ( Ouzzani et al., 2016).
