# Supplementary material for: Greenspace exposure and associated health outcomes: a systematic review of reviews
Source: F1000Res. 2025 Jul 30;13:491. Originally published 2024 May 17. [Version 2] doi: 10.12688/f1000research.148878.2 (PMC12426596; doi:10.12688/f1000research.148878.2)
Supplement: Peer review history

# Reviewer report: Reviewer response for version 2

**DOI:** 10.5256/f1000research.185657.r401603

**Published:** 2025-09-25

**Authors:** William Mueller

**Affiliations:** 1 Institute of Occupational Medicine (IOM), Edinburgh, UK

Thank you for providing the author responses to my comments. I am satisfied they have sufficiently addressed my points.

Are the rationale for, and objectives of, the Systematic Review clearly stated?

Yes

Is the statistical analysis and its interpretation appropriate?

Yes

If this is a Living Systematic Review, is the ‘living’ method appropriate and is the search schedule clearly defined and justified? (‘Living Systematic Review’ or a variation of this term should be included in the title.)

Not applicable

Are sufficient details of the methods and analysis provided to allow replication by others?

Yes

Are the conclusions drawn adequately supported by the results presented in the review?

Partly

Reviewer Expertise:

Environmental and occupational epidemiology

I confirm that I have read this submission and believe that I have an appropriate level of expertise to confirm that it is of an acceptable scientific standard.

# Reviewer report: Reviewer response for version 2

**DOI:** 10.5256/f1000research.185657.r408332

**Published:** 2025-09-11

**Authors:** David Rojas-Rueda

**Affiliations:** 1 Colorado State University, Fort Collins, USA

MAJOR COMMENTS

 1. Critically Outdated Search Strategy

 The search strategy, which ended in January 2021 (over four years ago), represents a fundamental limitation that significantly undermines the review's validity and applicability. This is particularly problematic for greenspace research given:

- Rapid expansion of high-quality research post-COVID-19
- Multiple systematic reviews published 2021-2024
- Evolution of methodological standards and exposure assessment techniques
- New evidence on mechanisms and dose-response relationships

Recommendation: Conduct a comprehensive updated search through 2025.

 2. Title Lacks Methodological Clarity

 The title "systematic review of reviews" should explicitly identify this as an umbrella review using standard terminology.

 Recommendation: Revise the title to include the term "umbrella review." 

 3. Critical Methodological Flaw in Meta-analysis Assessment

 Table 5 fails to report the study design composition of included meta-analyses, which is a fundamental methodological oversight. Many meta-analyses inappropriately pool cross-sectional studies with longitudinal studies, which:

- Violates basic meta-analysis principles
- Cannot establish causality
- Produces effect estimates with limited validity for causal inference
- Significantly affects evidence interpretation

Recommendation: Add columns to Table 5 showing:

- Number of studies in each meta-analysis
- Study design composition (# cross-sectional, # cohort, # RCT, etc.)
- Assessment of methodological appropriateness of pooling strategies
- Heterogeneity statistics (I²)

4. Quality Assessment Implications Inadequately Addressed

 With 50% of reviews having a high risk of bias and 36% rated as low quality, the implications for evidence reliability are inadequately discussed.

 Recommendation:

- Provide a detailed analysis of which AMSTAR-2 criteria were most commonly failed.
- Conduct sensitivity analyses excluding low-quality reviews
- Discuss how quality limitations affect confidence in conclusions

5. Limited Incremental Value Over Existing Reviews

 The comparison with existing umbrella reviews (van den Bosch & Ode Sang 2017; Yang et al. 2021) raises questions about unique contributions.

 Recommendation: More clearly articulate what this review adds beyond existing umbrella reviews, particularly given the temporal limitations.

 MINOR COMMENTS

 6. Methodological Transparency Issues

- Search Strategy: Include full search strategy in main text or easily accessible supplementary material
- Inter-rater Reliability: Provide statistics for study selection and quality assessment agreement
- Grey Literature: Clarify if grey literature was considered and justify exclusion if applicable

7. Results Presentation Problems

- PRISMA Flowchart: Reference is unclear - ensure easy accessibility or include key elements in the main manuscript
- Effect Size Reporting: Standardize decimal places and clarify whether estimates are from random or fixed effects models

8. Quantitative Synthesis Limitations

- Many meta-analyses based on very few studies (n=2-4)
- Missing heterogeneity assessments
- No discussion of the potential for publication bias potential
- Mixing of different exposure metrics in a single meta-analyses

9. ICD-10 Classification Concerns

 The post-hoc assignment of ICD-10 codes (Table 4) may not reflect how original studies defined outcomes and could be misleading.

 Recommendation: Remove or clearly label as authors' interpretation rather than original study classifications.

 10. Discussion Deficiencies

- Mechanistic Pathways: Brief mention of mechanisms could be expanded
- Policy Implications: Insufficient discussion of urban planning and public health implications
- Geographic Limitations: Most studies from high-income countries inadequately addressed
- Equity Considerations: Missing discussion of greenspace access disparities

11. Blue Space Exclusion Rationale

 While focusing solely on greenspace, the exclusion of studies examining combined green-blue space effects may artificially limit the evidence base and miss important findings.

 12. Temporal Analysis Missing

 No consideration of temporal trends in research quality, methodology, or findings over the study period.

 SPECIFIC TECHNICAL RECOMMENDATIONS

1. Restructure Results: Organize findings by strength of evidence rather than just outcome categories
2. Study Design Stratification: Re-analyze findings separately for longitudinal/cohort studies, cross-sectional studies, and intervention studies
3. Sensitivity Analyses: Exclude low-quality reviews to assess robustness of conclusions
4. Enhanced Quality Discussion: Provide a systematic analysis of methodological failures in included reviews
5. Future Research Priorities: More clearly identify evidence gaps and research priorities
6. Practical Implications: Expand discussion of what constitutes "effective" greenspace exposure for policy purposes

OVERALL ASSESSMENT

 This umbrella review addresses an important public health topic but suffers from significant methodological limitations. The outdated search strategy, inadequate assessment of meta-analysis quality, high proportion of low-quality included reviews, and presentation challenges substantially reduce its impact and reliability. The manuscript requires major revision addressing these fundamental issues before being suitable for publication in a high-impact journal.

 Recommendation: Major revision required, with particular attention to updating the evidence base, critically evaluating meta-analysis methodology, and enhancing transparency of quality assessment implications.

Are the rationale for, and objectives of, the Systematic Review clearly stated?

Partly

Is the statistical analysis and its interpretation appropriate?

Partly

If this is a Living Systematic Review, is the ‘living’ method appropriate and is the search schedule clearly defined and justified? (‘Living Systematic Review’ or a variation of this term should be included in the title.)

Not applicable

Are sufficient details of the methods and analysis provided to allow replication by others?

Partly

Are the conclusions drawn adequately supported by the results presented in the review?

Partly

Reviewer Expertise:

Environmental epidemiology

I confirm that I have read this submission and believe that I have an appropriate level of expertise to confirm that it is of an acceptable scientific standard, however I have significant reservations, as outlined above.

# Reviewer report: Reviewer response for version 2

**DOI:** 10.5256/f1000research.185657.r401604

**Published:** 2025-08-23

**Authors:** Wenzhong Huang

**Affiliations:** 1 Monash University, Melbourne, Australia

I appreciate the authors' efforts. The manuscript is now much clearer and improved over previous versions, particularly in clarifying its unique strengths and the comparisons with similar studies. The results have also been enhanced. I have no further comments.

Are the rationale for, and objectives of, the Systematic Review clearly stated?

Yes

Is the statistical analysis and its interpretation appropriate?

Partly

If this is a Living Systematic Review, is the ‘living’ method appropriate and is the search schedule clearly defined and justified? (‘Living Systematic Review’ or a variation of this term should be included in the title.)

Not applicable

Are sufficient details of the methods and analysis provided to allow replication by others?

Yes

Are the conclusions drawn adequately supported by the results presented in the review?

Yes

Reviewer Expertise:

Environmental epidemiolgy; greenness, air pollution, climate change and health

I confirm that I have read this submission and believe that I have an appropriate level of expertise to confirm that it is of an acceptable scientific standard.

# Reviewer report: Reviewer response for version 1

**DOI:** 10.5256/f1000research.163252.r284715

**Published:** 2024-06-28

**Authors:** William Mueller

**Affiliations:** 1 Institute of Occupational Medicine (IOM), Edinburgh, UK

Overall

 This is a well-conducted and well-written review about greenspace exposure and human health, a very active research area that would benefit from a broad synthesis of the evidence. I have outlined my comments below, which are mostly minor, but I do note an important Major comment for the authors and editor’s consideration.

 Major

 Much research on greenspace and health, including reviews, has been published since December 2020. If an updated review is underway, wouldn’t it be more valuable to wait to publish the more up to date work? Also given the reviews themselves have earlier cut-offs, so the current review would already be quite out of date with the most recent research.

 Minor

1. Consider changing title to refer to an ‘umbrella review’
2. Abstract - The abstract states that beneficial effects were observed for cardiovascular disease mortality, but that ambivalent results were found for cardiovascular health. The former is a subset of the latter, so please change wording to be consistent. [I see later that cardiovascular and metabolic health was combined as one category, but this is not clear from a read of the abstract.]
3. Abstract – it is always helpful to include some quantitative results, even if ranges of effect sizes, to better characterise findings
4. Introduction – ‘These studies did not consider overall health impacts’ – what does this statement mean exactly? Rephrase to make clear that full weight of evidence needs to be assessed to understand the nature of greenspace and health via an umbrella review, as described in the following sentence.
5. Introduction – Following the above point, what about reviews that address multiple health outcomes (e.g., Twohig-Bennett & Jones, 2018) [REF1]? Can they not provide useful information on overall health impacts as well?
6. Methods – Why are cardiovascular and metabolic outcomes combined?
7. Results – Did you search the references of included papers? If so, were any additional papers identified? Typically, reviews include flow charts to show the start and end number of papers.
8. Table 1- Why is the sample size for Carver et al. ‘NA’?
9. Table 1 – Define ‘NA’ with other short-forms
10. Table 3 – Add a ‘*’ or similar next to ‘Other’ to indicate that the specific exposures are given below.
11. Results – ‘Maternal health…’ – Change to ‘OR=0.80…’ for consistency.
12. Results – ‘Maternal health…’ – What do the Lee et al βs represent?
13. Table 5 – Title should refer to ‘meta-analyses’, otherwise it is not clear how the effect estimates were calculated. It would also be helpful to add the increment that the estimates represent (e.g. per 0.1 increase in NDVI)
14. Figure 1 – The methods should state how the overall risk of bias was determined (e.g., highest individual risk?). Also, the text accompanying the Figure, ‘…lighter colours concern judgments’ is not clear. Are these the individual risk of bias criteria? If so, state that.
15. Results – Risk of bias – why was the risk in some cases ‘unclear’? Was there not enough information to determine if a criterion was met? Wouldn’t that suggest it was not met and that it is likely to be a source of bias? This point should be considered.
16. Discussion – A stronger conclusion should be reached other than there may be an association between greenspace and health. For example, there appears to be stronger evidence for mental health, all-cause and CVD mortality. Are the specific pathways to health offered by greenspace more relevant for these outcomes?
17. Table 6 – Without knowing the AMSTAR-2 questions or how they were rated, this table is not very intuitive. The other tables are informative and effective at summarising the reviews and results.
18. Discussion – discussion of study limitations should be mentioned.

Are the rationale for, and objectives of, the Systematic Review clearly stated?

Yes

Is the statistical analysis and its interpretation appropriate?

Yes

If this is a Living Systematic Review, is the ‘living’ method appropriate and is the search schedule clearly defined and justified? (‘Living Systematic Review’ or a variation of this term should be included in the title.)

Not applicable

Are sufficient details of the methods and analysis provided to allow replication by others?

Yes

Are the conclusions drawn adequately supported by the results presented in the review?

Partly

Reviewer Expertise:

Environmental and occupational epidemiology

I confirm that I have read this submission and believe that I have an appropriate level of expertise to confirm that it is of an acceptable scientific standard, however I have significant reservations, as outlined above.

## References

[1] : The health benefits of the great outdoors: A systematic review and meta-analysis of greenspace exposure and health outcomes. *Environ Res* .2018;166: 10.1016/j.envres.2018.06.030 628-637 10.1016/j.envres.2018.06.030 29982151 PMC6562165

# Response

**Published:** 2025-07-25

**Authors:** Darsy Darssan

**Affiliations:** The University of Queensland, Australia

**Response to reviewer comments**

 We sincerely appreciate the thorough and insightful feedback on the manuscript. We have carefully considered and incorporated your suggestions wherever feasible.

**Major**

**Much research on greenspace and health, including reviews, has been published since December 2020. If an updated review is underway, wouldn’t it be more valuable to wait to publish the more up to date work? Also given the reviews themselves have earlier cut-offs, so the current review would already be quite out of date with the most recent research.**

 Response:

 As per the Cochrane guidelines (Cumpston and Flemyng, 2023), we have updated the review due to “the availability of new evidence that would have a meaningful impact on the review findings”. [ https://doi.org/10.12688/f1000research.166852.1].

 Cumpston, M., Flemyng, E., Chapter IV: Updating a review [last updated August 2023]. In: Higgins, J. P. T., Thomas, J., Chandler, J., Cumpston, M., Li, T., Page, M. J., Welch, V. A. (editors). *Cochrane Handbook for Systematic Reviews of Interventions *version 6.5. Cochrane, 2024. Available from https://www.cochrane.org/authors/handbooks-and-manuals/handbook/current/chapter-iv

 This review includes 36 systematic reviews published between January 2010 and December 2020. As you mentioned, much research on greenspace and health, including reviews, has been published since December 2020. We identified an additional 45 review articles between December 2020 and June 2024. Combining these two umbrella reviews would be too many systematic reviews to include in a single umbrella review.

**Minor**

**1. Consider changing title to refer to an ‘umbrella review’**

 In the title, we prefer to use the term ‘systematic review of reviews’, but we do refer to it as an umbrella review in the text. We find this title more understandable for a lay audience.

**2. Abstract - The abstract states that beneficial effects were observed for cardiovascular disease mortality, but that ambivalent results were found for cardiovascular health. The former is a subset of the latter, so please change wording to be consistent. [I see later that cardiovascular and metabolic health was combined as one category, but this is not clear from a read of the abstract.]**

 We have changed the wording in the abstract. In the results section, cardiovascular and metabolic health are reported under one category, but the results of the two outcomes are not combined.

**3. Abstract – it is always helpful to include some quantitative results, even if ranges of effect sizes, to better characterise findings**

 We have thought this through carefully and have concluded that synthesising the quantitative results could lead to bias, as we would be pooling the already pooled results from the systematic reviews. As per Aromataris et al. 2015 our umbrella review is intended to summarise the current evidence rather than re-synthesising the data.  

 Aromataris, E., Fernandez, R., Godfrey, C. M., Holly, C., Khalil, H., & Tungpunkom, P. (2015). Summarizing systematic reviews: methodological development, conduct and reporting of an umbrella review approach. *JBI Evidence Implementation*, *13*(3). https://doi.org/10.1097/XEB.0000000000000055  

**4. Introduction – ‘These studies did not consider overall health impacts’ – what does this statement mean exactly? Rephrase to make clear that full weight of evidence needs to be assessed to understand the nature of greenspace and health via an umbrella review, as described in the following sentence.**

 We reworded this section of the Introduction for better understanding.

**5. Introduction – Following the above point, what about reviews that address multiple health outcomes (e.g., Twohig-Bennett & Jones, 2018) [REF1]? Can they not provide useful information on overall health impacts as well?** 

 We reworded this section and have referenced Twohig-Bennett & Jones et al. 2018 in this section of the introduction.

**6. Methods – Why are cardiovascular and metabolic outcomes combined?**

 Cardiovascular and metabolic outcomes share common pathophysiological pathways and risk factors, making it logical to categorise them together. Greenspace exposure is hypothesised to influence these outcomes through shared mechanisms.

**7. Results – Did you search the references of included papers? If so, were any additional papers identified? Typically, reviews include flow charts to show the start and end number of papers.**

 Manual reference search is described in the ‘Search strategy’ section of the methods. As per the F1000 guidelines, a PRISMA flowchart for this review is available under the ‘Data availability’ -> ‘Reporting guidelines’ section at the end of the article.

**8. Table 1- Why is the sample size for Carver et al. ‘NA’?**

 This has been fixed to show the minimum and maximum sample size for Carver et al. 2020.

**9. Table 1 – Define ‘NA’ with other short-forms**

 This has been added.

10. Table 3 – Add a ‘*’ or similar next to ‘Other’ to indicate that the specific exposures are given below.

 This has been adjusted.  

**11. Results – ‘Maternal health…’ – Change to ‘OR=0.80…’ for consistency.**

 This has been adjusted.

**12. Results – ‘Maternal health…’ – What do the Lee et al βs represent?**

 The definition has been added to the first instance of β.

**13. Table 5 – Title should refer to ‘meta-analyses’, otherwise it is not clear how the effect estimates were calculated. It would also be helpful to add the increment that the estimates represent (e.g. per 0.1 increase in NDVI)**

 This has been adjusted. Also, we thoroughly reviewed this Table.

**14. Figure 1 – The methods should state how the overall risk of bias was determined (e.g., highest individual risk?). Also, the text accompanying the Figure, ‘…lighter colours concern judgments’ is not clear. Are these the individual risk of bias criteria? If so, state that.**

 ROBIS was used to assess the risk of bias of each systematic review. ROBIS consists of four domains, and within each domain, there are five to six signalling questions. Two reviewers went through each signalling question, and judgments were made independently. These domains were then used to determine the overall risk of bias of each review. Conflicts between the reviewers were resolved through discussion with the last author. For each review, the final decision on four domains and the overall risk of bias is listed in Supplementary Table S2. Figure 1 is a summary of Supplementary Table S2, as per ROBIS guidelines. More information about ROBIS is available from Whiting et al. 2016.

 Whiting, P., Savovic, J., Higgins, J. P., Caldwell, D. M., Reeves, B. C., Shea, B., Davies, P., Kleijnen, J., Churchill, R., & group, R. (2016). ROBIS: A new tool to assess risk of bias in systematic reviews was developed. *Journal of Clinical Epidemiology*, *69*, 225-234. https://doi.org/10.1016/j.jclinepi.2015.06.005  

 We have altered the Figure caption to improve the readability of the graph.

**15. Results – Risk of bias – why was the risk in some cases ‘unclear’? Was there not enough information to determine if a criterion was met? Wouldn’t that suggest it was not met and that it is likely to be a source of bias? This point should be considered.**

 As per the guidance on how to use ROBIS, an ‘unclear’ rating indicates that insufficient data were reported to permit a judgment for signalling questions within a domain (Phase 2).

 If something is not reported, it could be a potential source of bias. That’s why ROBIS has the unclear category, because if the information isn’t reported, you cannot make a definitive statement about whether the bias is high or low.

**16. Discussion – A stronger conclusion should be reached other than there may be an association between greenspace and health. For example, there appears to be stronger evidence for mental health, all-cause and CVD mortality. Are the specific pathways to health offered by greenspace more relevant for these outcomes?**

 In this narrative synthesis, stronger conclusions for each health outcome could lead to overstatements of the associations. Inconsistent findings within each health outcome, with varying effect measures, also make it challenging to report definitive conclusions.

 Some pathways may be more related to specific health outcomes and have been explored in more depth by other researchers (Markevych et al., 2017). However, these biological mechanisms are beyond the scope of this Discussion section.

 Markevych, I., Schoierer, J., Hartig, T., Chudnovsky, A., Hystad, P.,…. & Fuertes, E. (2017). Exploring pathways linking greenspace to health: Theoretical and methodological guidance. Environmental Research, 158, 301-317. https://doi.org/10.1016/j.envres.2017.06.028

**17. Table 6 – Without knowing the AMSTAR-2 questions or how they were rated, this table is not very intuitive. The other tables are informative and effective at summarising the reviews and results.**

 We have added the shortened AMSTAR-2 questions as a footnote to the table. The other reviewer also mentioned this table and we have coloured the overall quality column to improve readability. A graph showing the distribution of high, moderate, and low-quality reviews is also available in the supplementary material.

**18. Discussion – discussion of study limitations should be mentioned.**

 The limitations of the review have been discussed in the last paragraph of the discussion. We revisited this paragraph to improve the clarity.

# Reviewer report: Reviewer response for version 1

**DOI:** 10.5256/f1000research.163252.r284710

**Published:** 2024-06-10

**Authors:** Wenzhong Huang

**Affiliations:** 1 Monash University, Melbourne, Australia

**mandatory reviewer questions**

 Is the topic of the review discussed comprehensively in the context of the current literature?

 Yes

 Are all factual statements correct and adequately supported by citations?

 Mostly

 Is the review written in accessible language?

 Yes

 Are the conclusions drawn appropriate in the context of the current research literature?

 Yes

**Specific comments:**

 This study is a well-written umbrella review that synthesized systematic reviews on the associations of greenness and all kinds of health outcomes. I appreciate the great amount of work by the authors. I believe this study does have some value and can advance knowledge in this field. However, given the similarities to recent work by Stier-Jarmer et al. (2021)[Ref 1] and Yang et al. (2021)[Ref 2], I had the following suggestions/comments in an attempt to enhance the significance of this study.

 Introduction

 I appreciate the authors' introduction of two highly relevant umbrella reviews that incorporate the latest research advancements. Highlighting the advances of this study compared to these two reviews is crucial to justify its significance. However, the clarity of the following points could be improved: For instance, the review by van den Bosch and Ode Sang (2017) [Ref 3] includes both green and blue spaces, suggesting their work is more comprehensive than reviews focusing solely on green spaces. Therefore, it seems illogical for the authors to claim that the conclusions drawn from this study do not adequately represent the impact of green space exposure. Regarding the high-impact umbrella review by Yang et al., the authors mention a limitation involving the grouping of cancer and respiratory mortality into the section of ”other health outcomes”. This grouping likely resulted from the small number of studies on these topics and should not lead to outcome misclassification. Because Yang et al. could still discuss the findings for cancer and respiratory mortality separately. Additionally, including systematic reviews with variables such as physical activity, time spent outdoors, prosocial behavior, and murder as health outcomes does not appear to be a limitation. These reviews should encompass the health effects of greenness, (e.g., studies investigating the association of green spaces with health and the role of physical activity on the associations). In summary, the limitations mentioned by the authors are unclear and illogical to me. I understand and appreciate that this work possesses four important qualities compared to previous studies, as clearly mentioned in the last sentence of the Introduction. However, the significance of this research would be greatly enhanced if the authors provided clearer clarifications regarding the mentioned limitations of previous studies and how this study advances the field by addressing those limitations.

 Methods

 "We restricted the search to peer-reviewed systematic reviews and meta-analyses conducted in humans and published in English between January 2010 and December 2020, including reviews first published online during this period". Given the dramatic increase in publications on greenness and health in recent years, the quality of this study would be substantially improved if the authors included up-to-date reviews (i.e., from 2010 to the present, rather than 2010-2020). This inclusion can be mentioned as a major strength compared to previous umbrella reviews, as many high-quality studies and reviews have been published in the past three years.

 Full names should be provided for the abbreviations when they first appear in the main text (e.g., BMI, PA).

 Results

 It would be beneficial to include a flowchart illustrating the process used to select the final 36 systematic reviews (e.g., the number of studies excluded due to each specific exclusion criterion).

 It might be better to visualize the study design, measures of greenness, location of studies (country/territory), and outcomes in a figure rather than in several separate tables. A good example can be seen in https://link.springer.com/article/10.1007/s40572-023-00420-9 (Figure 2)

 Table 4. I appreciate the authors' usage of ICD-code to define the outcomes. However, if I understand it correctly, the original studies in these included reviews may not necessarily and consistently define their outcomes based on these ICD codes, right? If so, this table might be misleading.

 Why not combine Table 5 and Table 1, they have duplicated information.

 "Half of the systematic reviews (50%) had a high risk of bias“. This proportion is quite high. It would be better for the authors to clarify how they assigned the level of ROB to each of the reviews (e.g., using a table with the same structure as Table S2 to list the reason why each level was assigned). This was just a recommendation, but at least the authors should provide more clarifications and discuss this point. Currently, there are very few words on it.

 Consistent with the previous comment, a relatively high proportion of the included reviews were also classified as low quality. Providing more clarifications on this point would make this study stronger. Table 6, please clarify the meanings of these words (e.g., N, PY, NA) in the table caption. It would be better to assign the cell background with a color to indicate the quality level (e.g., red for low quality, green for high quality). This can improve the readability dramatically.

 Discussion

 "Additionally, we observed positive associations between greenspace exposure and maternal health and birth outcomes including PTB and SGA which were not present in other studies (Yang et al., 2021).” These observed positive associations between greenspace exposure and maternal health and birth outcomes including PTB and SGA were not that consistent compared to the outcomes of mental health and cognitive function, right? If so, this may be the reason why previous studies hadn't reported them in the main findings.

 "Conversely, Stier-Jarmer et al. (2021) and Yang et al. (2021) reported beneficial effects of greenspace on cardiovascular health and cardiometabolic factors, respectively, which was not replicated in our study.” This may be attributable to the fact that some reviews on this aspect were excluded in this umbrella review, while included in Stier-Jarmer et al. (2021) and Yang et al. (2021) due to the different inclusion and exclusion criteria (e.g., scoping reviews on greenness and CVD). Anyway, this should be discussed.

 "Current evidence, including this umbrella review, have been unable to establish a causal link between greenspace exposure and health outcomes as most studies are observational. This highlights the need for additional experimental studies to be conducted” Population-based experimental studies are often unfeasible and also subject to numerous biases (e.g., usually very small sample size, non-adherence). Given the very large proportion of cross-sectional studies, it is more practical to suggest more studies with longitudinal or cohort design and causal inference models (e.g., Instrumental Variables Analysis).

 "Most systematic reviews included in this review were limited by their methodological quality and/or risk of bias, and systematic reviews on the association between greenspace exposure and health outcomes have multiplied since 2020.” I am confused. The authors only included reviews between 2010-2020 and did not include reviews after 2020, as stated in the method section.

Are the rationale for, and objectives of, the Systematic Review clearly stated?

Yes

Is the statistical analysis and its interpretation appropriate?

Partly

If this is a Living Systematic Review, is the ‘living’ method appropriate and is the search schedule clearly defined and justified? (‘Living Systematic Review’ or a variation of this term should be included in the title.)

Not applicable

Are sufficient details of the methods and analysis provided to allow replication by others?

Yes

Are the conclusions drawn adequately supported by the results presented in the review?

Yes

Reviewer Expertise:

Environmental epidemiolgy; greenness, air pollution, climate change and health

I confirm that I have read this submission and believe that I have an appropriate level of expertise to confirm that it is of an acceptable scientific standard, however I have significant reservations, as outlined above.

## References

[1] : The Psychological and Physical Effects of Forests on Human Health: A Systematic Review of Systematic Reviews and Meta-Analyses. *Int J Environ Res Public Health* .2021;18(4) : 10.3390/ijerph18041770 10.3390/ijerph18041770 33670337 PMC7918603

[2] : Greenspace and human health: An umbrella review. *Innovation (Camb)* .2021;2(4) : 10.1016/j.xinn.2021.100164 100164 10.1016/j.xinn.2021.100164 34622241 PMC8479545

[3] : Urban natural environments as nature-based solutions for improved public health - A systematic review of reviews. *Environ Res* .2017;158: 10.1016/j.envres.2017.05.040 373-384 10.1016/j.envres.2017.05.040 28686952

# Response

**Published:** 2025-07-25

**Authors:** Darsy Darssan

**Affiliations:** The University of Queensland, Australia

**Response to reviewer comments**

 We are grateful for the valuable and detailed comments on our manuscript. We have thoughtfully reviewed and integrated your recommendations to the extent possible.

**Introduction**

**I appreciate the authors' introduction of two highly relevant umbrella reviews that incorporate the latest research advancements. Highlighting the advances of this study compared to these two reviews is crucial to justify its significance. However, the clarity of the following points could be improved: For instance, the review by van den Bosch and Ode Sang (2017) [Ref 3] includes both green and blue spaces, suggesting their work is more comprehensive than reviews focusing solely on green spaces. Therefore, it seems illogical for the authors to claim that the conclusions drawn from this study do not adequately represent the impact of green space exposure.**

 Our introduction does not claim that it inadequately represents the impact of greenspace exposure; rather, it assesses a composite of both greenspace and blue space effects on health. We have also amended the sentence to make this clearer.

**Regarding the high-impact umbrella review by Yang et al., the authors mention a limitation involving the grouping of cancer and respiratory mortality into the section of ”other health outcomes”. This grouping likely resulted from the small number of studies on these topics and should not lead to outcome misclassification. Because Yang et al. could still discuss the findings for cancer and respiratory mortality separately.**

 We have removed the section about cancer and respiratory health from this sentence.

**Additionally, including systematic reviews with variables such as physical activity, time spent outdoors, prosocial behavior, and murder as health outcomes does not appear to be a limitation. These reviews should encompass the health effects of greenness, (e.g., studies investigating the association of green spaces with health and the role of physical activity on the associations). In summary, the limitations mentioned by the authors are unclear and illogical to me.**

 Per the World Health Organization (WHO), “The determinants of health include: the social and economic environment, the physical environment, and the person’s individual characteristics and behaviours” (AIHW, 2024). Physical activity is classified as a behaviour (Chevance et al., 2019), making it a health determinant.

 Health determinants, such as physical activity or diet, contribute to health outcomes; however, as explained in our methods section, our review excluded studies that only considered health determinants.

 As you mention, physical activity plays a role in the association between greenspace and health outcomes (Chen et al., 2021), but it is not a health outcome itself. In our discussion, we explore physical activity as a pathway between greenspace exposure and health outcomes.

 World Health Organization (WHO). (2024). Determinants of health. https://www.who.int/news-room/questions-and-answers/item/determinants-of-health

 Chevance, G., Bernard, P., Chamberland, P. E., & Rebar, A. (2019). The association between implicit attitudes toward physical activity and physical activity behaviour: a systematic review and correlational meta-analysis. *Health Psychology Review*, *13*(3), 248–276. https://doi.org/10.1080/17437199.2019.1618726

 Chen, K., Zhang, T., Liu, F., Zhang, Y., & Song, Y. (2021). How Does Urban Green Space Impact Residents' Mental Health: A Literature Review of Mediators. *International journal of environmental research and public health*, *18*(22), 11746. https://doi.org/10.3390/ijerph182211746

 Australian Institute of Health and Welfare. (2024). *Social determinants of health.* Retrieved from https://www.aihw.gov.au/reports/australias-health/social-determinants-of-health  

 We revisited this section of the Introduction to improve the clarity.

**I understand and appreciate that this work possesses four important qualities compared to previous studies, as clearly mentioned in the last sentence of the Introduction.**

 Thank you, these four qualities are a major strength of this research.

**However, the significance of this research would be greatly enhanced if the authors provided clearer clarifications regarding the mentioned limitations of previous studies and how this study advances the field by addressing those limitations.**

 The limitations of the previous studies are:

 1. Do not isolate the impact of greenspace exposure alone on health outcomes, as its effects are confounded by blue space.

 2. Isolated impact of greenspace exposure on health outcomes alone

 3. Included scoping reviews in their umbrella review.

 To address these limitations, our review assesses the association between greenspace exposure and health outcomes, independent of blue space and health determinants. As per Pollack et al. 2023 and Aromataris 2015, an umbrella review should only include systematic reviews and meta-analyses.  Therefore, we excluded scoping reviews.

 Pollock, M., Fernandes, R. M., Becker, L. A., Pieper, D., Hartling, L. Chapter V: Overviews of Reviews [last updated August 2023]. In: Higgins, J. P. T., Thomas, J., Chandler, J., Cumpston, M., Li, T., Page, M. J., Welch, V. A. (editors). *Cochrane Handbook for Systematic Reviews of Interventions *version 6.5. Cochrane, 2024. Available from www.training.cochrane.org/handbook.

 Aromataris, E., Fernandez, R., Godfrey, C. M., Holly, C., Khalil, H., & Tungpunkom, P. (2015). Summarizing systematic reviews: methodological development, conduct and reporting of an umbrella review approach. *JBI Evidence Implementation*, *13*(3). https://doi.org/10.1097/XEB.0000000000000055  

**Methods**

**"We restricted the search to peer-reviewed systematic reviews and meta-analyses conducted in humans and published in English between January 2010 and December 2020, including reviews first published online during this period". Given the dramatic increase in publications on greenness and health in recent years, the quality of this study would be substantially improved if the authors included up-to-date reviews (i.e., from 2010 to the present, rather than 2010-2020). This inclusion can be mentioned as a major strength compared to previous umbrella reviews, as many high-quality studies and reviews have been published in the past three years.**

 This review includes 36 systematic reviews published between January 2010 and December 2020. As you mentioned, much research on greenspace and health, including reviews, has been published since December 2020. We identified an additional 45 review articles between December 2020 and June 2024. Combining these two umbrella reviews would be too many systematic reviews to include in a single umbrella review.

 As per the Cochrane guidelines (Cumpston and Flemyng, 2023), we have updated the review due to “the availability of new evidence that would have a meaningful impact on the review findings”. [Please see our updated review here, https://doi.org/10.12688/f1000research.166852.1].

 Cumpston, M., Flemyng, E., Chapter IV: Updating a review [last updated August 2023]. In: Higgins, J. P. T., Thomas, J., Chandler, J., Cumpston, M., Li, T., Page, M. J., Welch, V. A. (editors). *Cochrane Handbook for Systematic Reviews of Interventions *version 6.5. Cochrane, 2024. Available from https://www.cochrane.org/authors/handbooks-and-manuals/handbook/current/chapter-iv

**Full names should be provided for the abbreviations when they first appear in the main text (e.g., BMI, PA).**

 This has been fixed.

**Results**

**It would be beneficial to include a flowchart illustrating the process used to select the final 36 systematic reviews (e.g., the number of studies excluded due to each specific exclusion criterion).**

 As per the F1000 guidelines, a PRISMA flowchart for this review is available under the ‘Data availability’ à ‘Reporting guidelines’ section at the end of the article.

**It might be better to visualize the study design, measures of greenness, location of studies (country/territory), and outcomes in a figure rather than in several separate tables. A good example can be seen in https://link.springer.com/article/10.1007/s40572-023-00420-9 (Figure 2)**

 We agree that visualising these tables would be useful. However, the example given here, Figure 2, may be misleading. For instance, Figure 2 suggests that all ecological studies investigate respiratory outcomes; however, this is not the case, as indicated in Table S6 of the example.

 We attempted to create multi-level pie charts to visualise our Tables but found that this was too crowded and inefficient. After several attempts, we decided not to present these multi-level pie charts.

**Table 4. I appreciate the authors' usage of ICD-code to define the outcomes. However, if I understand it correctly, the original studies in these included reviews may not necessarily and consistently define their outcomes based on these ICD codes, right? If so, this table might be misleading.**

 You are correct that the original studies may not have defined the outcomes based on the ICD codes.

 Based on the information available in the reviews, we allocated all potential ICD codes for each health outcome. We did this to promote standardised reporting in future articles that investigate the association between greenspace exposure and health outcomes.

**Why not combine Table 5 and Table 1, they have duplicated information.**

 Table 5 summarises the quantitative results from the included reviews and does not duplicate information from Table 1.

**"Half of the systematic reviews (50%) had a high risk of bias“. This proportion is quite high. It would be better for the authors to clarify how they assigned the level of ROB to each of the reviews (e.g., using a table with the same structure as Table S2 to list the reason why each level was assigned). This was just a recommendation, but at least the authors should provide more clarifications and discuss this point. Currently, there are very few words on it.**

 ROBIS consists of four domains, and within each domain, there are five to six signalling questions. Two reviewers went through each signalling question, and judgments were made independently. These domains were then used to determine the overall risk of bias of each review. Conflicts between the reviewers were resolved through discussion with the last author. For each review, the final decision on four domains and the overall risk of bias is listed in Supplementary Table S2. More information about ROBIS is available from Whiting et al. 2016.

 Whiting, P., Savovic, J., Higgins, J. P., Caldwell, D. M., Reeves, B. C., Shea, B., Davies, P., Kleijnen, J., Churchill, R., & group, R. (2016). ROBIS: A new tool to assess risk of bias in systematic reviews was developed. *Journal of Clinical Epidemiology*, *69*, 225-234. https://doi.org/10.1016/j.jclinepi.2015.06.005 

**Consistent with the previous comment, a relatively high proportion of the included reviews were also classified as low quality. Providing more clarifications on this point would make this study stronger. Table 6, please clarify the meanings of these words (e.g., N, PY, NA) in the table caption. It would be better to assign the cell background with a color to indicate the quality level (e.g., red for low quality, green for high quality). This can improve the readability dramatically.**

 We have added the meanings of the AMSTAR abbreviations and have added colour to the overall quality column to increase readability. We have also added a footnote explaining the AMSTAR-2 questions, so the reader understands how the quality rating was decided.

**Discussion**

**"Additionally, we observed positive associations between greenspace exposure and maternal health and birth outcomes including PTB and SGA which were not present in other studies (Yang et al., 2021).” These observed positive associations between greenspace exposure and maternal health and birth outcomes including PTB and SGA were not that consistent compared to the outcomes of mental health and cognitive function, right? If so, this may be the reason why previous studies hadn't reported them in the main findings.**

 We revised this sentence.

**"Conversely, Stier-Jarmer et al. (2021) and Yang et al. (2021) reported beneficial effects of greenspace on cardiovascular health and cardiometabolic factors, respectively, which was not replicated in our study.” This may be attributable to the fact that some reviews on this aspect were excluded in this umbrella review, while included in Stier-Jarmer et al. (2021) and Yang et al. (2021) due to the different inclusion and exclusion criteria (e.g., scoping reviews on greenness and CVD). Anyway, this should be discussed.**

 We revised this sentence.

**"Current evidence, including this umbrella review, have been unable to establish a causal link between greenspace exposure and health outcomes as most studies are observational. This highlights the need for additional experimental studies to be conducted” Population-based experimental studies are often unfeasible and also subject to numerous biases (e.g., usually very small sample size, non-adherence). Given the very large proportion of cross-sectional studies, it is more practical to suggest more studies with longitudinal or cohort design and causal inference models (e.g., Instrumental Variables Analysis).**

 We agree that population-based experimental studies are rare. We have revised this sentence accordingly.

**"Most systematic reviews included in this review were limited by their methodological quality and/or risk of bias, and systematic reviews on the association between greenspace exposure and health outcomes have multiplied since 2020.” I am confused. The authors only included reviews between 2010-2020 and did not include reviews after 2020, as stated in the method section.**

 We revised this sentence
